# Validity and Reliability of the Heart Rate Matching Task: A Novel Measure of Heart Rate Estimation

**DOI:** 10.1111/psyp.70284

**Published:** 2026-04-02

**Authors:** Jamie A. Moffatt, Markus R. Tünte, Mariana Von Mohr, Manos Tsakiris

**Affiliations:** ^1^ Lab of Action & Body, Department of Psychology Royal Holloway, University of London Egham England; ^2^ Department of Psychology Durham University Durham England; ^3^ Department of Developmental and Educational Psychology University of Vienna Vienna Austria

**Keywords:** body perception, interoception, measurement, online research, reliability, validity

## Abstract

Sensing and monitoring changes in our heart rate is a key aspect of interoception. We introduce and test the construct validity of the Heart Rate Matching task (HRM), a novel, fast and accessible task designed to assess the ability to estimate heart rate, in both remote (i.e., online) and in‐lab settings. In the first lab experiment, under‐ and over‐estimations of the heart rate were not significantly correlated with two interoceptive tasks designed to assess perception of individual heartbeats; the adapted Heartbeat Counting and the Multi‐Interval Heartbeat Discrimination tasks. A second experiment conducted online in a large sample, found moderate significant correlations between Heart Rate Matching and the original Heartbeat Counting task. The HRM also correlated with a matched exteroceptive task, highlighting involvement of shared multi‐sensory integration processes. The third experiment demonstrated good test–retest reliability of the HRM and moderate correlations with the Heart Rate Discrimination task, also thought to assess the estimation of heart rate. Taken together, these data suggest acceptable validity and moderate reliability of the Heart Rate Matching task and fit with an interpretation of the task as a measure of heart rate beliefs.

## Impact Statement

The Heart Rate Matching task (HRM) is a novel measure of cardiac interoceptive beliefs. Across three experiments, we report good construct validity of the task, meaning researchers can use it with confidence. Importantly, the HRM is accessible, quick and engaging, meaning it is particularly suited for researchers with strict time requirements or limited access to specialist cardiac recording equipment.

## 1 Introduction

A quickly beating heart following exercise is a sure sign of the intensity of the workout, whereas entraining a slower, calmer heart rate is the goal for precision sports like archery or shooting. An accurate ability to monitor one's internal bodily signals not only confers an advantage in sporting contexts (Li et al. 2021), but also in the day‐to‐day monitoring of one's own health and the regulation of emotions (Füstös et al. 2013). The sensing, interpretation and integration of signals from within the body is known as interoception (Desmedt, Luminet, Maurage, et al. Desmedt, Luminet, Maurage, and Corneille 2023; Khalsa et al. 2018). Interoceptive ability can be assessed across multiple dimensions, from the biological quality of afferent signals from the nervous system, to the neural representation of those signals, to the sensing of those signals and then to the higher‐level beliefs and interpretation of interoceptive signals (Suksasilp and Garfinkel 2022). At the behavioral level, much interoceptive research seeks to measure how well individuals can objectively detect bodily signals like the heartbeat, termed interoceptive accuracy, while others assess interoceptive beliefs such as one's estimation in how quickly the heart is beating (Legrand et al. 2022). Here, we present the Heart Rate Matching task, a novel cardiac interoception measure, and three experiments which seek to determine which aspect of interoception the task measures, as well as its construct validity.

The Heartbeat Counting task (Schandry 1981), the most widely used interoception measure, assesses how well participants can count their heartbeats over time. While often labeled as an interoceptive accuracy measure (e.g., Garfinkel et al. 2015), the Heartbeat Counting task arguably measures both perceptual accuracy and prior held beliefs about the heart: good performance can come from either being able to perceive each heartbeat or from having prior knowledge of the pace of one's own heart rate. For those seeking to measure interoceptive accuracy, the confounding influence of beliefs is a severe limitation of the task. In recognition of this limitation, some suggest the Heartbeat Counting task should only be used with adapted instructions to encourage counting of only explicitly felt heartbeats (Desmedt et al. 2018). Others recommend using alternative tasks, like the Heartbeat Discrimination task (Katkin et al. 1981; Whitehead et al. 1977), which asks participants to judge if exteroceptive (typically auditory) stimuli is presented either in‐ or out of time with the heartbeat, a design which removes the confounding influence of heart rate beliefs. Psychophysical versions of the task such as the Method of Constant Stimuli (Brener et al. 1993), where sequences of tones are presented at multiple intervals from the peak of the heartbeat signal, have been labeled as the most robust measures of interoceptive accuracy (Brener and Ring 2016). However, these approaches can be lengthy and require specialist equipment to precisely time the heart signal and present stimuli with millisecond precision.

The limitations of Heartbeat Counting and Discrimination tasks have led to the recent development of several new measures of cardiac interoceptive accuracy (e.g., Herman et al. 2021; Larsson et al. 2021; Maister et al. 2017; Plans et al. 2021; Ponzo et al. 2021; Smith et al. 2021). For example, in the Phase Adjustment Task (PAT; Plans et al. 2021) participants align auditory tones with their heartbeats, with their real heartbeats detected by smartphone camera‐driven photoplethysmography, meaning the task is suitable for remote testing (Spooner et al. 2024). The PAT demonstrated moderate test–retest reliability, but its internal reliability (e.g., split‐half assessment) and convergent validity with other behavioral tasks are yet to be determined, a limitation of many of the new measures of interoceptive accuracy (for a review, see Desmedt, Luminet, Walentynowicz, et al. Desmedt, Luminet, Walentynowicz, and Corneille 2023).

An alternative response to the limitations of tasks assessing accuracy in sensing heartbeats is to explicitly measure beliefs about heart rate. Measuring interoceptive beliefs, rather than interoceptive accuracy, may be important for a number of reasons. First, it is a noted limitation that only a small portion of participants can detect heartbeats at rest. This is not a criticism of interoceptive accuracy tasks, but likely reflects the true proportion of the population who can detect their heartbeat at rest (Brener et al. 1993; Körmendi et al. 2023; Plans et al. 2021). In contrast, heart rate beliefs can be assessed in all people. Second, certain clinical groups appear to differ in heart rate beliefs; estimations of heart rate were more inaccurate in people with a diagnosis of schizophrenia compared to a nonclinical group (Jeganathan et al. 2025). Third, an expanding area of interoception research concerns how visceral sensations are interpreted. For example, individuals may vary how they appraise and attribute causes of a rising or falling heart rate. When studying these higher‐order facets of interoception, the extent to which an individual accurately perceives their heartbeat may be less relevant than what an individual believes about their heart rate (Savage and Garfinkel 2025). Finally, the development of novel measures of heart rate beliefs also aligns with recent calls to move beyond measuring interoceptive accuracy in interoception research, given the methodological difficulties with its measurement (Desmedt and Van den Bergh 2024; Körmendi et al. 2023; Murphy 2024).

One task which targets measurement of interoceptive beliefs is the Heart Rate Discrimination task (HRD; Legrand et al. 2022), which asks participants to determine if sequences of auditory tones are slower or faster than their own heart rate. Bayesian psychophysical modeling allowed researchers to determine the bias (under‐ or over‐estimation of heart rate), as well as the precision (uncertainty of estimate) of heart rate beliefs. Estimates of bias were found to have good test–retest reliability and only a small correlation with bias estimates from an exteroceptive control task, suggesting good discriminant validity (Legrand et al. 2022). However, the task is difficult for researchers and clinicians to implement quickly and at scale. The task is lengthy, with the quickest version of the task involving 80 trials and lasting between a reported 18–32 min. In addition, because the adaptive psychophysical procedure requires real‐time recording and analysis of heart rate across short time (< 8 s) frames, application of the task requires specialist equipment such as electrocardiography or photoplethysmography.

In order to enable assessment of interoception rapidly and at scale, we present a newly developed measure of cardiac interoception, the Heart Rate Matching (HRM) task, designed to be a quick, accessible, and engaging method to measure the ability to monitor heart rate both remotely as well as in lab settings (freely available at Gorilla Open Materials, https://app.gorilla.sc/openmaterials/868003). The HRM uses a method of adjustment approach (Gescheider 2013), where participants view a pulsing cartoon heart and can speed up or slow down its pulsation. The participant must match the pace of the viewed heart with the pace of their own heartbeat. Averages of reported and actual heart rate are then compared to determine bias in heart rate beliefs, denoting the extent to which heart rate is under‐ or over‐estimated. The method of adjustment procedures encourage active participation throughout, which is considered to be more engaging for the participant (Gescheider 2013). In addition, the task is compatible with remote photoplethysmography (rPPG) assessments of heart rate, allowing for large‐scale online testing. rPPG describes algorithms which estimate heart rate based on fluctuations of color in the face associated with the cardiac cycle (van der Kooij and Naber 2019), and show good validity (Di Lernia et al. 2024) in estimating heart rate from webcam video recordings of the face. The HRM was adapted from an earlier task (Palmer et al. 2019) designed to measure interoceptive accuracy, yet similarly to Heartbeat Counting, it is likely to also be influenced by prior knowledge of the heart rate. To some degree, the HRM likely measures both interoceptive accuracy and interoceptive beliefs and we opted for a data‐driven approach to determine with which construct the task more closely aligns.

Across three experiments and three independent samples, we sought to compare performance on HRM with other interoceptive tasks to determine which aspect of interoception it more closely relates to: accuracy in perceiving heartbeats (interoceptive accuracy) or estimation of heart rate (interoceptive beliefs). In addition, we sought to assess the construct validity of the HRM. In the first experiment conducted with participants in person, we assessed the association of bias on the HRM with established methods for assessing interoceptive accuracy, the adapted Heartbeat Counting task (Desmedt et al. 2018) and a 6‐interval Heartbeat Discrimination task. In the second experiment conducted online in a larger sample, we tested associations between the HRM and performance on a matched exteroceptive control task, the Audio Rate Matching (ARM) task. In this larger sample, we also explored associations with the original Heartbeat Counting task and three interoceptive questionnaires, the Interoceptive Accuracy Scale (Murphy et al. 2020), the Interoceptive Attention Scale (Gabriele et al. 2022) and the Multi‐dimensional Assessment of Interoceptive Awareness (Mehling et al. 2018). In this experiment we also assessed the predictive validity of the HRM by assessing associations between HRM performance and constructs associated with interoception, such as alexithymia and anxiety, and reported the internal reliability of the HRM. In the third experiment conducted in person, we determined the test–retest reliability of the HRM and examined how performance on the task relates to performance on the Heart Rate Discrimination task, to determine how closely the HRM relates to a measure of cardiac interoceptive beliefs.

## 2 Methods—Experiment 1

### 2.1 Participants

Ethical approvals for Experiments 1, 2 and 3 were obtained from the University Research Ethics Committee at Royal Holloway, University of London prior to the onset of data collection. Sample size determination and recruitment for Experiment 1 are described elsewhere (Moffatt et al. 2024). Forty‐two participants were recruited to take part in Experiment 1 which involved two sessions, though only 41 completed the second session that is reported in this experiment. Sample size for this experiment was based on power for the previous study, but after data analysis we conducted a sensitivity analysis for a correlation in Gpower 3.1 software (Faul et al. 2007), which found that for a sample size *N* = 41, the study is powered to detect correlations of *r* = 0.38 with 80% power and *r* = 0.48 with 95% power.

### 2.2 Design

The experiment involved two sessions. In the first session, participants completed a rubber hand illusion task, the results of which are reported elsewhere (Moffatt et al. 2024). In the second session, participants completed three tasks designed to assess cardiac interoceptive ability. Participants completed the Heartbeat Counting task (Schandry 1981), the 6‐interval Heartbeat Discrimination task (Brener et al. 1993; Ring and Brener 2018), and the newly developed Heart Rate Matching task. The Discrimination task was always completed last because it provides information about the speed of the heartbeat to the participant, which could have influenced performance on the Counting and Matching tasks. The order of the Counting and Matching tasks was counterbalanced between participants. The Discrimination and Counting tasks were implemented in the Psychtoolbox extension of MATLAB (Version 3.0, Kleiner et al. 2007), the HRM task was implemented in Gorilla Experiment Builder.

#### 2.2.1 Heart Rate Matching (HRM)

**FIGURE 1:**
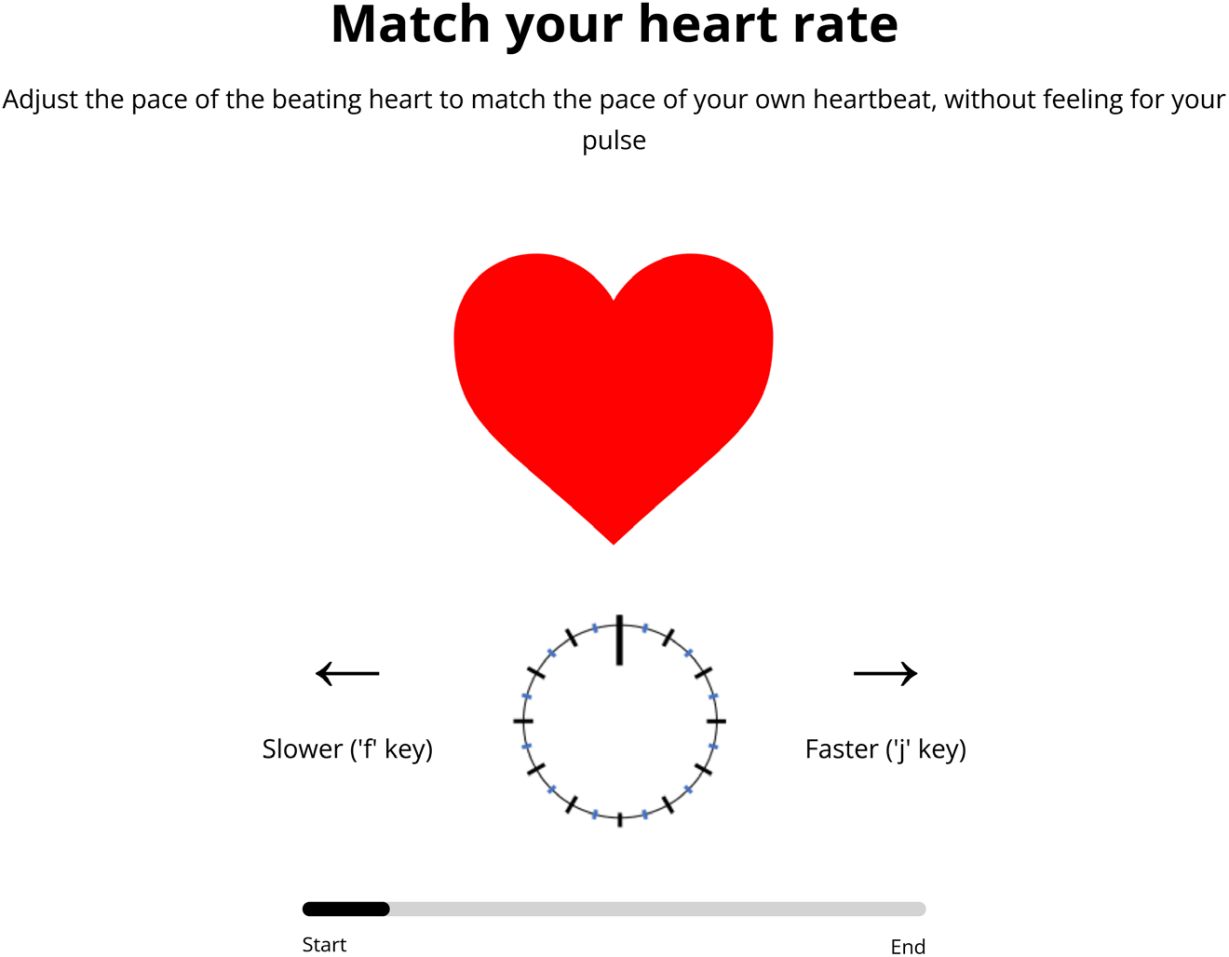
Display for Heart Rate Matching task.

The HRM task is a method of adjustment procedure (Gescheider 2013), adapted from an earlier task (Palmer et al. 2019). There were 6 trials in which participants saw an image of a cartoon heart. The cartoon heart regularly “pulsed” by increasing in size for 100 ms before decreasing to the normal size. During the trial, participants were asked to match the pace of the beating heart to the pace of their own heartbeat, without feeling for their pulse, and adjust the rate of the visual beating heart to match their own heartbeat. Pressing the ‘f’ key on the keyboard decreased the beats‐per‐minute (BPM) of the beating heart by 2 BPM and pressing the “j” key increased the BPM by 2 BPM, with a minimum of 30 BPM and a maximum of 130 BPM. To aid participants visualize the change in heart rate, a dial rotated clockwise as the heart sped up and counterclockwise as the heart slowed down (see Figure 1).

The starting pace of the cartoon heart was set to be either lower or higher than the population average resting heart rate of 68 BPM (Mason et al. 2007). The starting heart rate of “low” trials was drawn from a uniform random distribution between 20 and 30 BPM, whereas the starting heart rate of “high” trials was drawn from between 110 and 120 BPM. These values were chosen to be outside the range of typical resting heart rates, rather than personalized to each participant, to enable quick data collection. Only 3 participants had average resting heart rates greater than the lower bound of “high” trials (110 BPM). Participants completed an equal number of “low” and “high” trials in a random order. The duration of each trial was drawn from a uniform distribution between 35 and 40 s. A progress bar ensured participants knew how much time they had left for each trial. Participants could adjust the dial as much as they wished throughout the trial, and the pace of the cartoon heart at the end was taken as their reported BPM. After each trial, participants rated how confident they were that their response matched the pace of their own heartbeat, on a VAS from 0 (Not confident at all) to 100 (Extremely confident). Participants were also asked if they felt like they had enough time during the trial to provide a response (yes/no).

**FIGURE 2:**
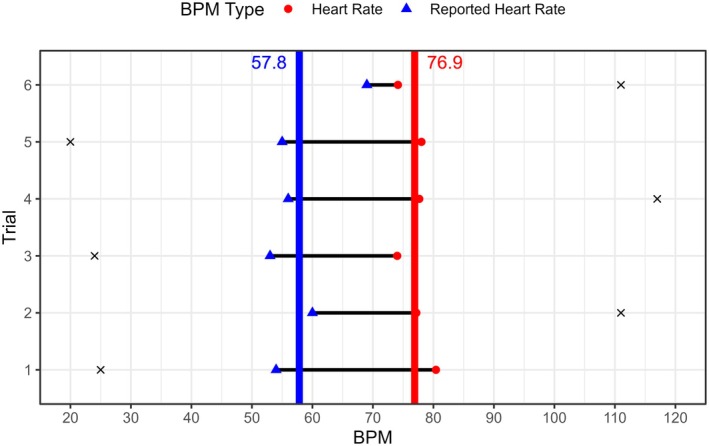
Heart Rate Matching data from exemplar participant. Each row is a trial of the HRM. Blue triangles denote reported heart rate, red circles denote estimated heart rate from electrocardiography recording, with the vertical lines and text denoting averages across all trials. Crosses represent the starting BPM of the visual heart on each trial. Bias is the difference between the red and blue vertical lines, Variance captures the amount the horizontal black lines differ across trials. BPM, Beats per minute.

The design of the HRM allows for the estimation of three key variables: *bias*, *variance* and *confidence*. Bias reflects how well an individual was able to estimate their heart rate. Bias was calculated as the discrepancy between the average heart rate and the average reported heart rate across trials (Figure 2). A negative bias represents an under‐estimation of heart rate, while a positive bias represents an over‐estimation of heart rate. Averaging across the “high” and “low” trials reduces the possibility for starting heart rate to influence bias estimates, and is standard practice with Method of Adjustment approaches (Gescheider 2013). In addition to bias, we calculated the absolute value of bias, to represent how accurate the heart rate estimate was regardless of under‐or‐over estimation. The second metric of variance captures the consistency of an individual's heart rate estimation, with lower values suggesting greater consistency in estimating heart rate, and higher values relating to lower consistency in estimating heart rate. Variance was calculated as the standard error of the differences between actual and reported heart rate. Finally, confidence in estimating heart rate was calculated as the average confidence rating across all trials.

### 2.3 Heartbeat Discrimination

A 6‐interval Heartbeat Discrimination procedure (Brener et al. 1993) was adapted for use in this study. On each trial, participants were presented with a series of 5 auditory tones delivered at either 0, 100, 200, 300, 400, or 500 ms following the peak of the R wave, with 20 sequences at each level of delay. The 120 trials were divided into five blocks of 24 trials, with each delay time occurring 4 times in a random order within each block. After listening to each sequence of tones, participants judged if the tones were in or out of time with their heartbeat and rated their confidence in their judgment on a continuous scale from 0 to 100. The outcome measure of interoceptive accuracy represents precision in detecting heartbeats and was calculated as the interquartile range (IQR) of the temporal distribution of “in‐time” judgments (Brener et al. 1993).

### 2.4 Heartbeat Counting

For the Heartbeat Counting task (Schandry 1981), participants were asked to count their heartbeats during three randomly ordered time periods (25 s, 35 s, 45 s). After each time period, they stated how often they had felt their heartbeat and provided a confidence rating on a continuous scale from 0 to 100. Prior to the task, participants were given clear instructions to report how many heartbeats they explicitly felt during the time period and to not guess their answer, in accordance with the adapted instructions of the Heartbeat Counting task (Desmedt et al. 2018) to reduce the influence of non‐interoceptive processes on the outcome measure. Interoceptive Accuracy was calculated by comparing reported and real heartbeats, with the following equation (Ainley et al. 2020):

$$\frac{1}{3}\sum \left(1-\frac{\left|\text{Actual heart beats}-\text{Reported heart beats}\right|}{\text{Actual heart beats}}\right)$$

### 2.5 Physiological Recordings

During all 3 tasks, heart recordings were taken using electrocardiography. Heart rate was recorded throughout the experiment with Electrocardiography (ECG), using three disposable ECG electrodes. An electrode was positioned underneath each collarbone and a third on the left side of the lower back. The ECG signal was recorded with a Powerlab 8/35 (Powerlab, ADInstruments, http://www.adinstruments.com/) using Labchart 8 Pro software. The sampling rate was 1000 Hz and a hardware band‐pass filter (Bio Amp 132) between 0.3 and 1000 Hz was applied.

For the HRM, video recordings of the face were recorded on each trial. Video recordings were taken using MediaStream Recording API implemented in JavaScript to record video from a webcam positioned at the top of the computer screen with > 20 frames per second. A softbox studio light positioned behind the screen ensured adequate luminance. Videos (640 × 480 resolution) were converted from VP80 codec and .webm to .mp4 format using HandBrake (https://handbrake‐fr/) with parameters Production Max, Quality Lossless (Placebo), with all other parameters set to default (Di Lernia et al. 2024). After conversion, videos were analyzed offline in MATLAB using remote photoplethysmography techniques (van der Kooij and Naber 2019) to estimate heart rate from facial color changes related to the flow of blood around the body.

### 2.6 Analysis

Outcome variables for the HRM, Heartbeat Counting, and Heartbeat Discrimination tasks were assessed for normality with Shapiro–Wilk tests. If a variable was non‐normally distributed, a log transformation was applied and assessed for normality. Pearson correlations were used to assess associations between variables. For calculation of all metrics, the ECG estimate of heart rate was used rather than the rPPG estimate of heart rate unless otherwise stated.

## 3 Results—Experiment 1

Participants had a mean age of 19.63 (SD = 2.32), with 37 identifying as Female (90.24%) and the remainder as Male. Heartbeat recordings for the Heartbeat Counting task were excessively noisy for 1 participant preventing accurate identification of the number of heartbeats from the ECG recording, and behavioral data failed to record due to a technical error for another, meaning *N* = 39 were included in analysis of the counting task. For the Heartbeat Discrimination task, an additional participant was excluded due to noisy heart recordings, meaning *N* = 39 were included in analyses of the Discrimination task. Time to complete the HRM task was 7.29 min on average (SD = 0.90).

Averages for the HRM are reported in Table 1. Bias scores (reported heart rate minus actual heart rate) were under‐estimated by 19.8 BPM on average. Average Heartbeat Counting score was 0.31 (SD = 0.22), similar to previous studies using these instructions (Desmedt et al. 2018), with confidence rated on average at 0.38 (SD = 0.23). On average, performance on the Heartbeat Discrimination task, calculated as the IQR of the cumulative frequency distribution of “synchronous” judgments, was 287.38 ms (SD = 34.36 ms), with a range of 207‐350 ms, with chi‐square tests of the distribution of simultaneous judgments revealing only 10% of participants responded differently from chance. The average IQR is higher (and therefore less precise) than previous studies (e.g., Ring and Brener 2018, mean = 265 ms, SD = 48 ms).

**TABLE 1:** Averages for the outcome variables for the matching tasks in Experiments 1, 2, and 3.

	*N*	Reported BPM	Target BPM	Bias (BPM)	Abs Bias (BPM)	Variance	Confidence
HRM – Study 1	41	68.6	88.3	−19.8	22.2	8.54	49.8
HRM – Study 2	95	64.69	73.72	−9.03	14.64	4.46	61.10
ARM – Study 2	131	75.80	69.41	6.38	7.70	6.54	77.81
HRM – Study 3, Session 1	34	82.9	82.7	0.23	12.99	8.58	55.9
HRM – Study 3, Session 2	34	83.9	82.9	1.02	14.96	7.1	55.6

Abbreviations: ARM, Audio Rate Matching; BPM, beats‐per‐minute; HRM, Heart Rate Matching; *N*, sample size.

All variables from the HRM were normally distributed according to Shapiro–Wilk tests, except for the Absolute Bias and Variance scores, therefore the log values of Absolute Bias and Variance were calculated with base 10, which were normally distributed. Both Interoceptive Accuracy derived from the Heartbeat Counting task (${\text{IAcc}}_{\mathrm{HBC}}$) and the Heartbeat Discrimination task (${\text{IAcc}}_{\mathrm{HBD}}$) were normally distributed.

### 3.1 Associations Between Different Variables of the HRM

**FIGURE 3:**
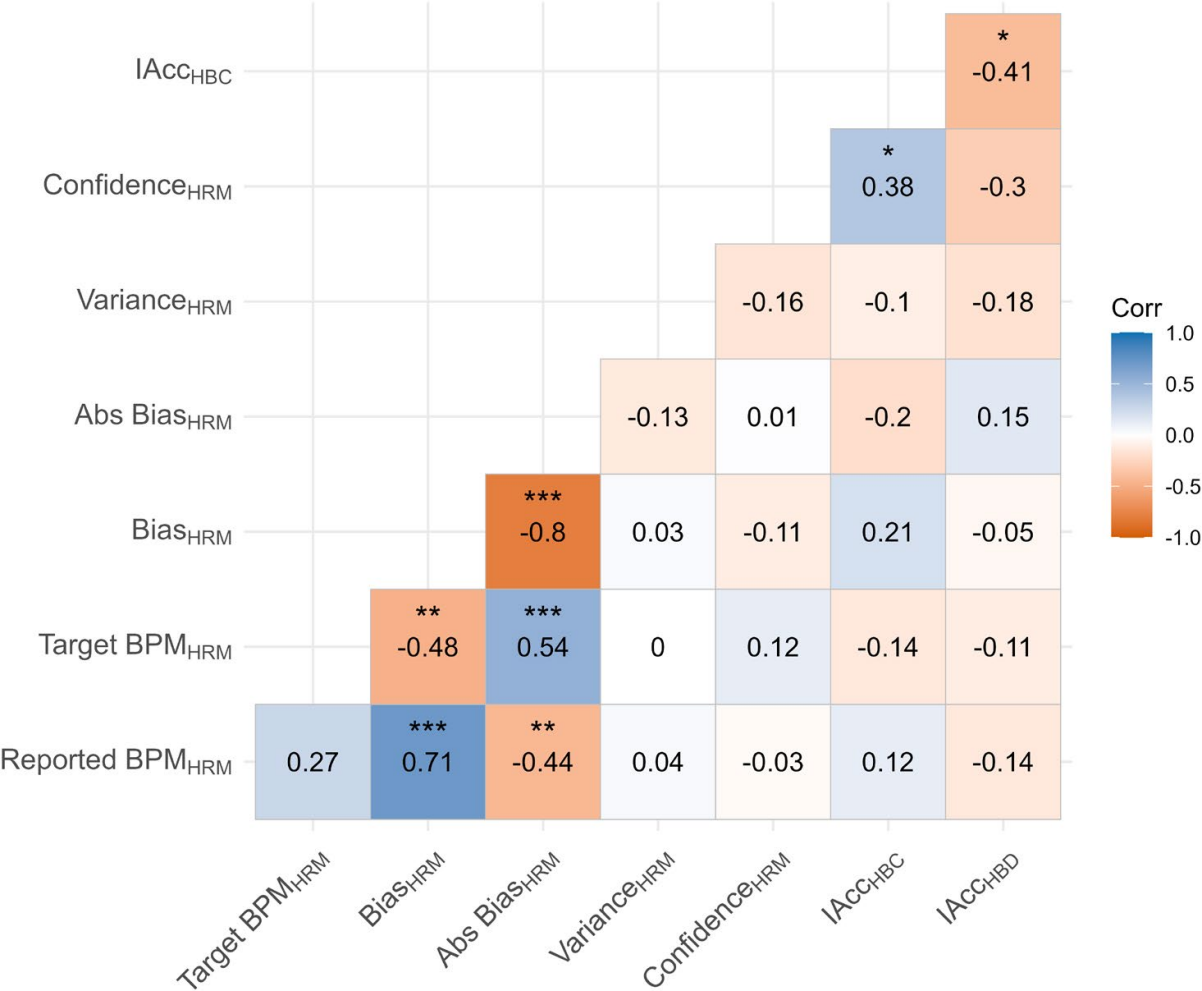
Associations between Matching, Counting and Discrimination tasks. Absolute Bias and Variance are log values. BPM, beats‐per‐minute; HBC, Heartbeat Counting; HBD, Heartbeat Discrimination; HRM, Heart Rate Matching; IAcc, Interoceptive Accuracy. IAcc for HBD is the inter‐quartile range of the cumulative frequency distribution of ‘synchronous’ judgments. IAcc for HBC is the reported heartbeats compared to actual heartbeats, averaged across trials. *p < 0.05, **p < 0.01, ***p < 0.001.

Bias score correlated significantly positively with reported BPM and negatively with target BPM (Figure 3). As reported BPM increased, Bias score was more likely to be reduced. In addition, as target BPM increased, Bias score was more likely to increase. These associations follow logically from the calculation of Bias, which is reported BPM subtracted from target BPM.

### 3.2 Associations Between Heart Rate Matching, Heartbeat Counting, and Heartbeat Discrimination

**FIGURE 4:**
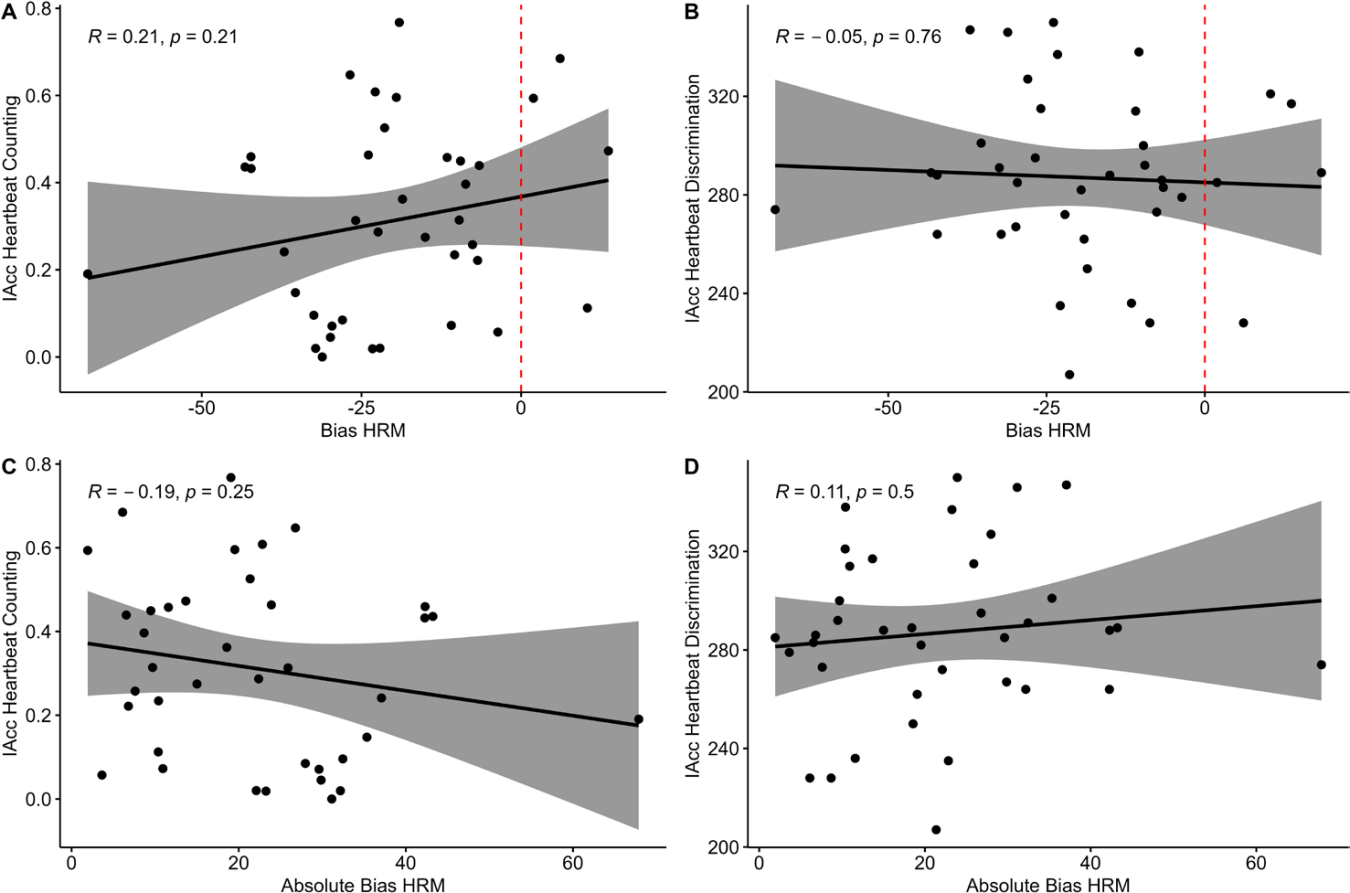
Associations between Bias and IAcc. (A) Correlation between IAcc derived from Heartbeat Counting task and Heart Rate Matching Bias. (B) Correlation between IAcc derived from Heartbeat Discrimination and Heart Rate Matching Bias. (C and D) are the same correlations as (A and B) but with the absolute value of Bias.

Bias was not significantly correlated with IAcc measured via either Heartbeat Counting or Heartbeat Discrimination IQR scores (Figure 4). ${\text{IAcc}}_{\mathrm{HBC}}$ was significantly positively correlated with ${\text{Confidence}}_{\mathrm{HRM}}$, but no other parameters of the HRM correlated significantly with IAcc from either task. The relation between HRM variables and IAcc are largely similar when HRM metrics are calculated with the rPPG estimate of heart rate (see Supporting Informations).

IAcc assessed with Heartbeat Counting and Heartbeat Discrimination was significantly correlated together, suggesting that the Heartbeat Discrimination and the Heartbeat Counting tasks measure related constructs.

### 3.3 Comparison of Heart Rate Estimation Methods

**FIGURE 5:**
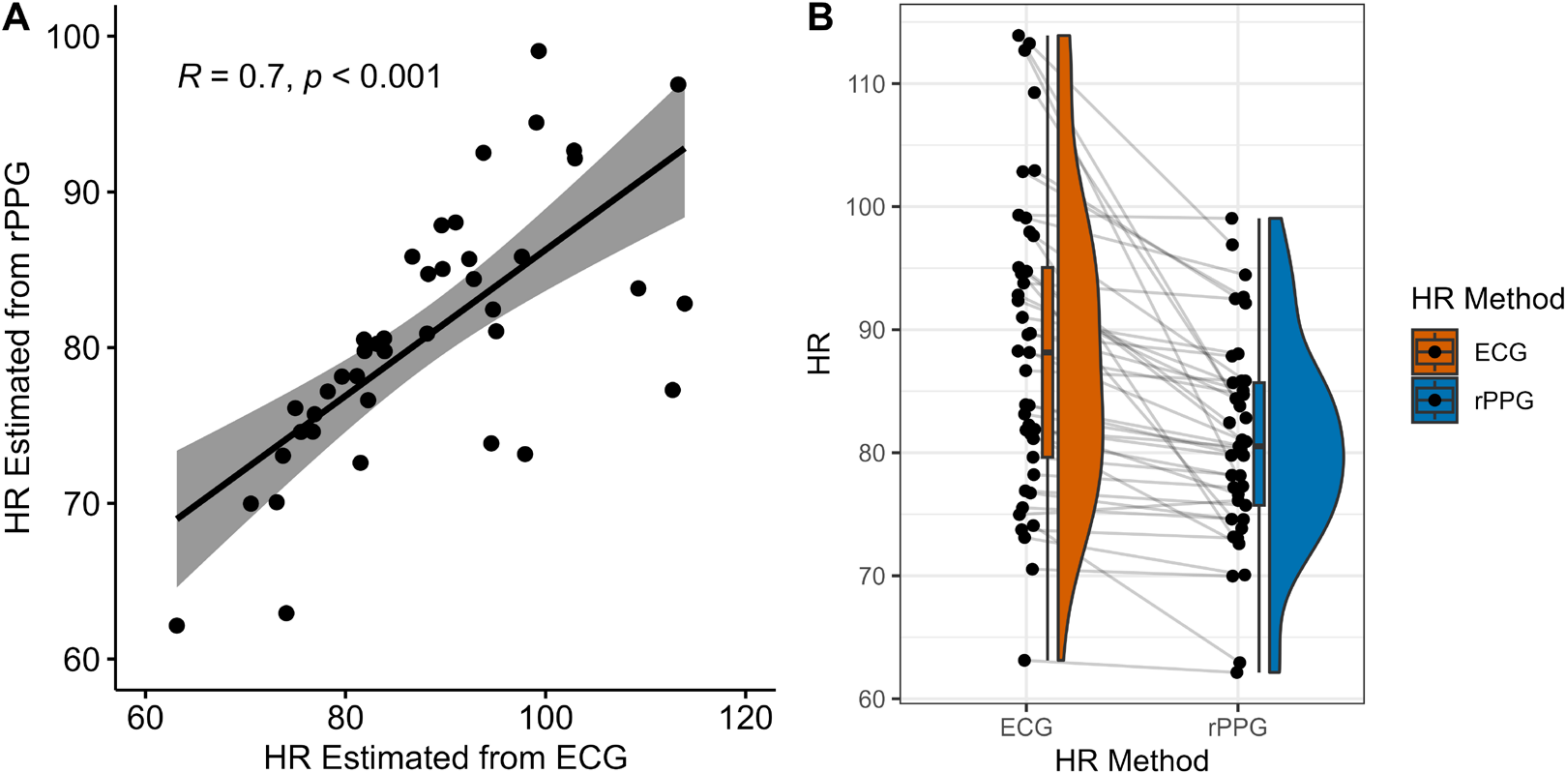
Comparison of Heart Rate (HR) estimation methods. (A) Correlation between HR estimated across trials of the Heart Rate Matching task from remote photoplethysmography (rPPG) and HR estimated from electrocardiography (ECG). (B) Difference in HR estimated from rPPG and ECG methods.

Finally, Experiment 1 offered a unique opportunity to compare heart rate estimated via ECG, the gold standard for recording cardiac activity, and rPPG methods. rPPG data was inspected for potential erroneous heart rates according to recommended criteria (Di Lernia et al. 2024). However, no videos had a frame rate below 20 frames‐per‐second, and all heart rates were estimated between 50 and 120 BPM. Although heart rates estimated from two videos from the same participant were flagged as outliers using the R boxplot function, their ECG heart rates were not flagged as outliers, and therefore these videos were retained. Heart rates estimated from ECG and rPPG were highly positively correlated when averaged per participant (*r* = 0.70, *p* < 0.001, 95% CI [0.49, 0.83], see Figure 5A). However, there were significant differences between heart rate estimated from ECG and Heart Rate estimated from rPPG (*t*(40) = 5.46, *p* < 0.001, 95% CI [4.74, 10.31]), with rPPG methods underestimating Heart Rate by 7.52 BPM on average (Figure 5B). The inaccuracy in estimated heart rate from rPPG propagates to Bias and Variance derived from the HRM using the rPPG estimate of heart rate, as the calculation of these metrics involve the estimate of heart rate. Bias is 7.52 BPM higher when using rPPG estimate (−12.25), which is significantly greater than bias calculated from ECG heart rate estimate (*t*(40) = −5.46, *p* < 0.001, 95% CI [−10.31, −4.38]), although the two estimates correlate closely (*r* = 0.87, p < 0.001, 95% CI [0.76, 0.93]). Variance estimated from rPPG and Variance estimated from ECG correlate extremely closely (*r* = 0.99, *p* < 0.001, 95% CI [0.986, 0.996]), but are significantly different (*t*(40) = −4.64, p < 0.001, 95% CI [−0.54, −0.21]). The discrepancy in heart rate estimates appears to be driven by inaccuracy in the rPPG for estimating higher heart rates. Participants with higher than the median heart rate (88.15 BPM) had an average discrepancy between rPPG and ECG heart rate of −12.1 BPM, whereas participants with lower than median heart rate had an average discrepancy of −2.7 BPM.

## 4 Discussion—Experiment 1

Using the HRM task, a novel method of determining ability to estimate heart rate, participants under‐estimated their heart rate by 19.8 BPM on average. This large under‐estimation bias was potentially driven by a particularly high average resting heart rate in this sample of 88.3 BPM. Nevertheless, the experiment demonstrated that interoceptive accuracy scores derived from widely used interoception tasks, the Heartbeat Discrimination task and the adapted Heartbeat Counting task, did not significantly correlate with outcome variables from the novel HRM task. The experiment also replicated previous findings that the rPPG assessment of heart rate derived from the recordings of faces is closely associated with heart rate derived from ECG, but the rPPG estimate was a significant 7.5 BPM lower on average compared to the ECG HR estimate. As noted previously, higher heart rates are less accurately estimated by this method (van der Kooij and Naber 2019), and we found evidence that the ECG‐rPPG discrepancy in heart rate was driven mainly by larger discrepancies in people with higher heart rates. rPPG is a topic of ongoing, active development (e.g., Di Lernia et al. 2024; Liu et al. 2023; Pirzada et al. 2023); therefore, we expect the performance of rPPG algorithms to improve in the near future. It should be noted that the relationships between HRM metrics and the two measures of Interoceptive Accuracy were highly similar regardless of whether HRM metrics were calculated with ECG or rPPG methodology (see Supporting Informations).

Since the HRM task did not correlate with two widely used tasks of interoceptive accuracy, it begs the question of what aspect of interoception the HRM measures. In Experiment 2, we tested how performance on the HRM task was associated with the original Heartbeat Counting task (Schandry 1981). The original version of the Heartbeat Counting task asks participants to count their heartbeats without any instructions to only count explicitly felt heartbeats. Therefore, participants can take alternative strategies to counting each individual heartbeat and may instead estimate their heart rate based on prior knowledge or beliefs. As the bias measure of the HRM is likely to also measure estimation of heart rate, there may be a significant correlation between the HRM and the original Heartbeat Counting task. In addition, we compared HRM performance to performance on a control task, the Audio Matching Task, in a test of discriminant validity. Finally, we further assessed convergent validity and predictive validity of the HRM by assessing how the HRM relates to self‐reported interoceptive ability and constructs related to interoception including anxiety, alexithymia, and dissociation. Given the demonstration in Experiment 1 that rPPG methods correlate closely with estimates of heart rate using EEG methods, Experiment 2 was conducted fully remotely online, allowing us to reach a larger sample of participants.

## 5 Methods—Experiment 2

### 5.1 Participants

rPPG performance drops significantly when attempting to estimate heart rate in people with darker skin tones (Nowara et al. 2020), therefore, we identified 509 participants with white ethnicity from a previous study and invited them to take part via Prolific. Of these 509, 153 participants completed the study, of which 149 passed at least 2/3 of embedded attention checks. Mean age was 47.83 (SD = 14.17), and 75 identified as Female (50.34%), with the remaining 74 identifying as Male (49.66%).

### 5.2 Design

All tasks were implemented in Gorilla Experiment Builder, and the questionnaires were completed in Qualtrics. Prior to both cardiac tasks, participants were told not to feel for their pulse.

### 5.3 Heart Rate Matching

The HRM was almost identical to Experiment 1, with the only change being that the number of trials was increased from 6 to 12 trials. No participants had an average heart rate greater than the lower bound of the starting BPM on “high” trials (110 BPM).

### 5.4 Heartbeat Counting

The counting task was almost identical to Experiment 1, with the only change being the instructions given to participants. The classic instructions were used (Schandry 1981), where participants were *not* told to only count explicitly felt heartbeats.

### 5.5 Audio Rate Matching

The Audio Rate Matching (ARM) task is an exteroceptive version of the HRM, designed to act as a control task. Instead of matching the visual beating heart to their own heart rate, participants match the pace of the visual beating heart to the rate of a repeating auditory tone across 12 trials. The ARM included “low” and “high” trials, identical to the HRM. The rate at which the tones played on a given trial was drawn from a random uniform distribution between 50 and 90 BPM. The final change from the HRM is that participants could adjust the visual beating heart and submit their answer whenever they wished, rather than having a fixed duration. The maximum time allocated was 2 min per trial, at which point the experiment moved to the next trial. A warning message to submit their response appeared 30 s before the maximum time.

### 5.6 Questionnaires

The Interoceptive Accuracy Scale (IAS) is a 21‐item scale assessing how accurate people feel they are at perceiving internal bodily sensations (Murphy et al. 2020). Each item is a statement about how accurately the individual can perceive a specific bodily sensation (e.g., “I can always accurately perceive when my heart is beating fast”) and is rated on a Likert scale from 1 (Disagree strongly) to 5 (Strongly agree). The IAS had a mean score of 82.01 (SD = 12.68) and Cronbach's Alpha of *α* = 0.93.

The Interoceptive Attention Scale (IATS) is a 21‐item scale assessing how much attention is paid to internal bodily sensations (Gabriele et al. 2022). Each item is a statement about how attentive an individual is to a specific bodily sensation (e.g., “Most of the time my attention is focused on whether my heart is beating fast”) and is rated on a likert scale from 1 (Disagree strongly) to 5 (Strongly agree). The IATS had a mean score of 54.66 (SD = 16.23) and *α* = 0.94.

The Multi‐dimensional Assessment of Interoceptive Awareness, Version 2 (MAIA‐2) is a 37‐item scale designed to assess interoceptive awareness (Mehling et al. 2012, 2018). Each item is a statement about bodily awareness (e.g., “When I am tense, I notice where the tension is located in my body”) and is rated on a Likert scale from 0 (Never) to 5 (Always). It is divided into eight subscales of Noticing (M = 3.37, SD = 0.98, *α* = 0.76), Not‐Distracting (M = 2.1, SD = 0.98, *α* = 0.84), Not‐Worrying (M = 2.59, SD = 0.96, *α* = 0.75), Attention Regulation (M = 2.98, SD = 1.06, *α* = 0.91), Emotional Awareness (M = 3.31, SD = 1.02, *α* = 0.83), Self‐Regulation (M = 3.05, SD = 1.17, *α* = 0.86), Body Listening (M = 2.92, SD = 1.16, *α* = 0.84), and Body Trust (M = 3.36, SD = 1.18, *α* = 0.82).

The Cambridge Depersonalization Scale (CDS) is a 29‐item scale which assesses the duration and frequency of depersonalization experiences (Sierra and Berrios 2000). Each item is rated on Frequency on a scale from 0 (Never) to 4 (All the Time) and Duration from 0 (a few seconds) to 6 (more than a week), which are summed. The CDS had a mean of 20.81, SD = 25.84, *α* = 0.92.

The Toronto Alexithymia Scale (TAS‐20) is a 20‐item scale assessing alexithymia (Bagby et al. 1994). Each item is a statement about emotional feeling (e.g., “I am often confused about what emotion I am feeling”), rated on a Likert scale from 1 (Strongly disagree) to 5 (Strongly agree). The TAS‐20 has a mean of 43.01, SD = 11.22, *α* = 0.88.

The State–Trait Inventory for Cognitive and Somatic Anxiety (STICSA) is a 21‐item scale assessing cognitive and somatic aspects of anxious feelings (Ree et al. 2008). There is a subscale of Cognitive Anxiety (M = 1.7, SD = 0.69, *α* = 0.92) and Somatic Anxiety (M = 1.34, SD = 0.47, *α* = 0.91). Each item is a statement (e.g., “I think the worst will happen”) rated on a Likert scale from 1 (“Almost Never”) to 4 (“Almost Always”).

### 5.7 Physiological Recordings

Heart rate during the trials of the Heartbeat Counting and HRM tasks was estimated from recorded videos of the face during the tasks. Videos were analyzed using rPPG methodology (van der Kooij and Naber 2019) to determine heart rate, as outlined in the methods for Experiment 1.

### 5.8 Procedure

After reading an information sheet and providing consent, participants completed a webcam check which determined if their webcam worked correctly. If their webcam did not work, they were provided some troubleshooting guidance (e.g., checking webcam was correctly plugged in). If the problems persisted, they were informed they could not take part and took no further part in the study. After passing the webcam check, participants viewed their own video in real time and were given some guidelines to follow to ensure that the video was usable for rPPG such as sitting in a lit area (Lernia et al. 2022). An auditory check was also completed to ensure that participants could correctly hear audio from the experiment. Across three trials, participants listened to a word and then chose which word was spoken from three options. If less than 2/3 words were correctly identified, the participant was informed that they could not take part and took no further part in the study. Only a single participant (of the 509 invited) was unable to take part due to webcam failure, no participants were rejected based on the auditory check (6.7% reported 2/3 words correct, 93.3% reported 3/3 words correct).

The Heartbeat Counting and HRM tasks were counterbalanced between participants, and the ARM task was always completed after the two cardiac tasks. After completing these tasks, participants were forwarded to a Qualtrics questionnaire with the CDS and the TAS‐20.

As this was the fourth wave of a previous study, participants had already provided demographic information and completed the IAS, IATS, MAIA, and STICSA questionnaires in previous waves. 824 participants completed Wave 1 between 4th and 7th November 2022, and 705 of those completed Wave 2 between 21st November and 6th December 2022. All questionnaire data reported in the present study were collected during Wave 3, which was completed between December 5th and 13th 2022. Wave 4, containing the HRM, ARM, Heartbeat Counting tasks and the CDS and TAS‐20 questionnaires, was completed between 6th January 2023 and 23rd March 2023.

## 6 Results—Experiment 2

### 6.1 rPPG Filtering

Following the same exclusion criteria as Experiment 1, and as used in previous research (Di Lernia et al. 2024), we excluded trials if the rPPG algorithm was unable to estimate heart rate, if the frame rate of the video was below 20FPS or if the estimated heart rate was outside of 50–120 BPM.

For the Heartbeat Counting task, for 16.8% of trials, the rPPG failed to extract heart rate from the video and for 21.2% of trials, the video had a frame rate below 20 Frames‐Per‐Second (FPS). No videos were excluded for falling outside of 50–120 BPM heart rate. In total, the videos of 61.9% of trials were retained.

For the HRM task, for 15.1% of trials, the rPPG failed to extract heart rate from the video; for 22.0% of trials, the video had a frame rate below 20 fps; and for 0.11% of trials, the estimated heart rate was outside the range of 50–120 BPM. In total, the videos of 62.9% of trials were retained.

### 6.2 Behavioral Filtering

Additional filtering was carried out for the Matching tasks to identify and remove trials associated with careless responding.

First, trials with too‐many or too‐few responses were identified—too few responses suggest a lack of engagement in the task, whereas too many button presses may reflect careless responding (e.g., excessively holding down a button). The mean number of button presses was 49.82 (SD = 55.88) for the heart task and 54.63 (SD = 35.71) for the audio task. Trials with button presses greater than 3 standard deviations than the mean were removed. For the HRM, this identified 5 trials from 2 participants. For the ARM, this identified 22 trials from 17 participants. Any trial with 5 or fewer button presses were also removed, which removed 27 trials from 18 participants from the HRM, and 50 trials from 32 participants from the ARM.

Second, for the auditory task where participants could respond whenever they wanted, mean submission time was 22.94 s, ranging from 2 to 120 s. We removed 39 trials from 24 participants who submitted their response in 5 s or less, as this may indicate careless responding.

Finally, we identified two participants with unusual button use in the ARM task, where they never increased the heart rate, with 100% of button presses across all trials making the heart rate slower. Since the matching tasks were designed to have some trials starting with a high BPM and other trials starting with a low BPM, use of only a single button across all trials makes little sense and suggests careless responding. Data on the ARM from these participants were excluded entirely. An additional participant was excluded from both matching tasks due to the BPM of the cartoon heartbeat not matching the heart rate set by the participant—an issue likely caused by poor internet connection speeds.

After excluding participants for the preceding reasons, and excluding those with fewer than 2 trials remaining for Heartbeat Counting (*N* = 8), and fewer than 2 trials remaining in both ‘high’ and ‘low’ conditions of the heart and audio matching tasks (*N* = 22), there were 94 participants remaining for the Heartbeat Counting, 95 for the HRM task and 131 for the ARM task. The 12‐trial version of the HRM took 11.43 min to complete on average (SD = 1.82), the ARM took 8.07 min (SD = 3.16), and the HBC took 4 min (SD = 1.67).

### 6.3 Normality

According to Shapiro–Wilk tests, Heartbeat Counting score was non‐normally distributed, as were reported BPM, confidence, and variance for the HRM task, as well as confidence and variance for the auditory task. Applying a log transformation to these variables did not influence the normality of the distributions, therefore nonparametric spearman correlations are reported for this data.

### 6.4 Heart Rate Matching

**FIGURE 6:**
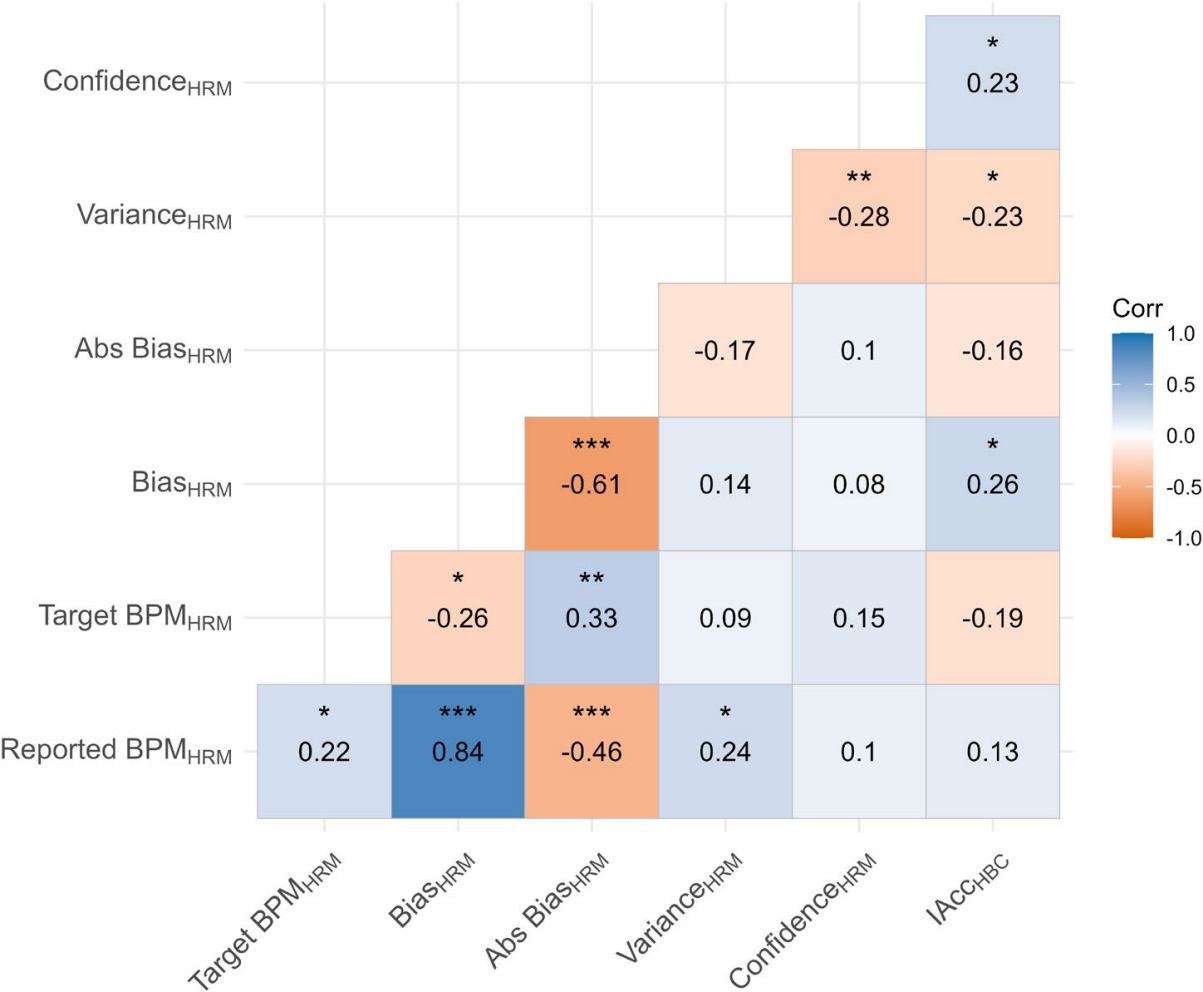
Associations between Heart Rate Matching and Heartbeat Counting. Statistic is Spearman correlation coefficient, *p < 0.05, **p < 0.01, ***p < 0.001. BPM, beats‐per‐minute; HBC, Heartbeat Counting; HRM, Heart Rate Matching.

In line with our findings from Experiment 1, bias was significantly positively correlated with reported BPM and significantly negatively correlated with target BPM (Figure 6). The correlation between reported and target BPM had a similar correlation coefficient to Experiment 1, but this time was significant. Variance was significantly negatively correlated with confidence ratings, suggesting that greater variance in response was related to lower confidence ratings. This correlation was not significant in Experiment 1.

### 6.5 Heart Rate Matching and Heartbeat Counting

Heartbeat Counting score was 0.69 on average (SD = 0.22) and Confidence was 0.54 on average (SD = 0.27), both notably higher than Experiment 1 which used the HBC with adapted instructions. This is consistent with previous studies comparing performance in both variants of the HBC (Desmedt et al. 2018).

**FIGURE 7:**
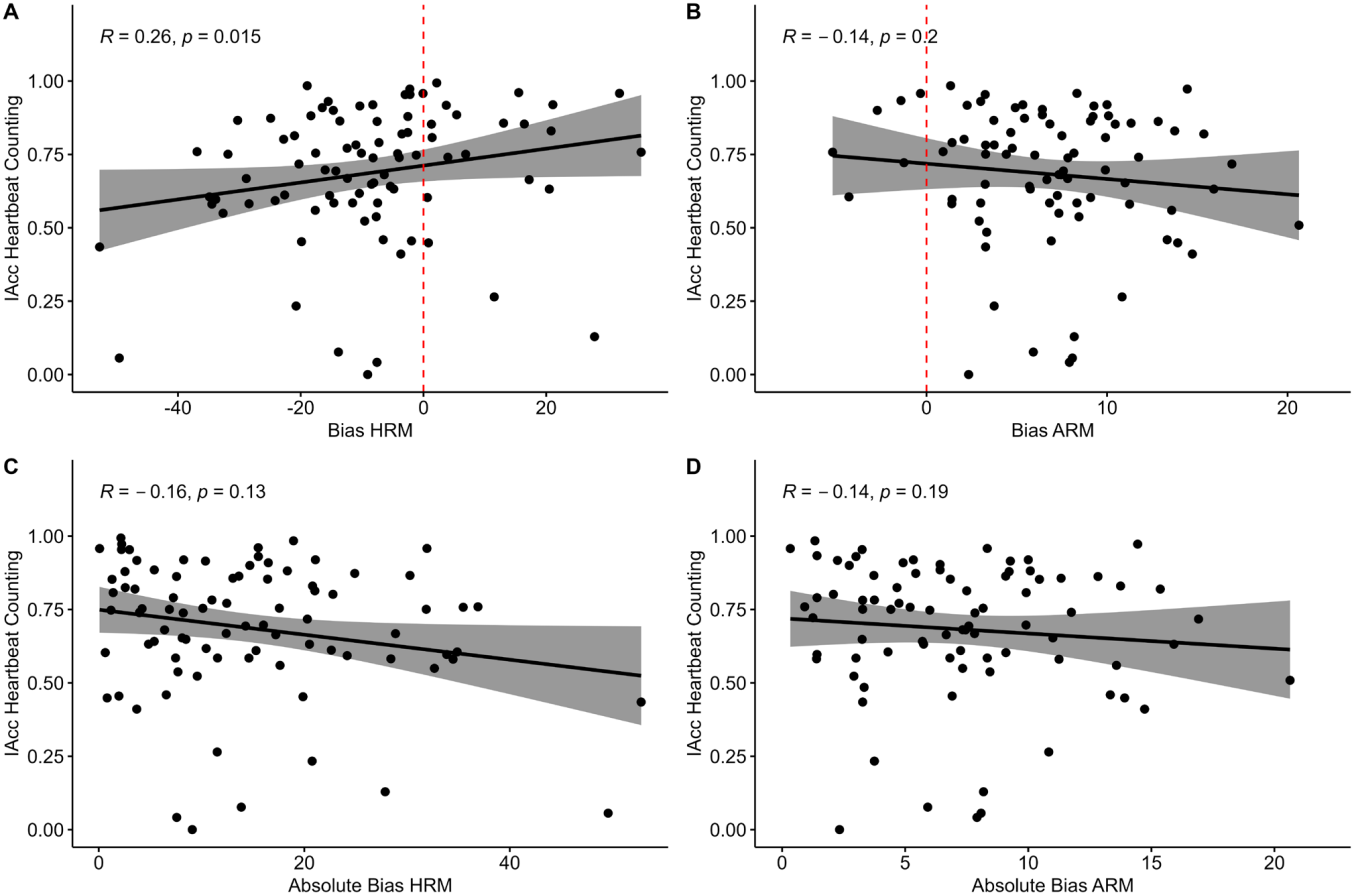
Association between Interoceptive Accuracy, HRM and ARM. (A) Correlation between HRM Bias and IAcc. (B) Correlation between ARM Bias and IAcc. (C) Correlation between IAcc and HRM Absolute Bias. (D) Correlation between IAcc and ARM Absolute Bias. Statistic is Spearman Correlation co‐efficient. ARM, Audio Rate Matching; HRM, Heart Rate Matching; IAcc, Interoceptive Accuracy.

To test convergent validity of the HRM, we tested the correlation between Heartbeat Counting score and the HRM task. In contrast to Experiment 1, Heartbeat Counting score was significantly positively correlated with bias on the HRM (*ρ* = 0.26, *p* = 0.015; Figures 6 and 7), such that higher Counting score was related to a more positive bias in estimating heart rate, and lower Counting score was related to a more negative bias. Heartbeat Counting score did not significantly correlate with absolute bias on the HRM (*ρ* = −0.16, *p* = 0.13), but was significantly correlated with both HRM Variance (*ρ* = −0.23, *p* = 0.03) and HRM Confidence (*ρ* = 0.23, *p* = 0.03). In addition, Heartbeat Counting did not significantly correlate with bias on the ARM (*ρ* = −0.14, *p* = 0.20; Figure 4B), nor with any other ARM variable. To test if correlations with the Heartbeat Counting task were driven by shared interoceptive processes, we used z‐tests to examine if correlations between the HRM and Heartbeat Counting were significantly different from the equivalent correlations between Heartbeat Counting and the ARM task. Only HRM confidence showed a significantly closer correlation with Heartbeat Counting score compared to ARM confidence (*z* = 2.03, *p* = 0.042, 95% CI [0.008, 0.438]), with neither the comparison of the Bias (*z* = 1.62, *p* = 0.11, 95% CI [−0.05, 0.503]) nor Variance (*z* = −1.657, *p* = 0.098, 95% CI [−0.455, 0.039]) reaching significance.

Actual heart rate estimated using rPPG methods from the Heartbeat Counting trials (Mean = 72.77 BPM) and heart rate estimated from the HRM trials (Mean = 73.72 BPM) at the subject level were significantly strongly correlated (*r* = 0.87, *p* < 0.001, 95% CI [0.81 0.92]), suggesting heart rate was consistently estimated with the rPPG methods across tasks.

### 6.6 Heart Rate Matching and Audio Rate Matching

**FIGURE 8:**
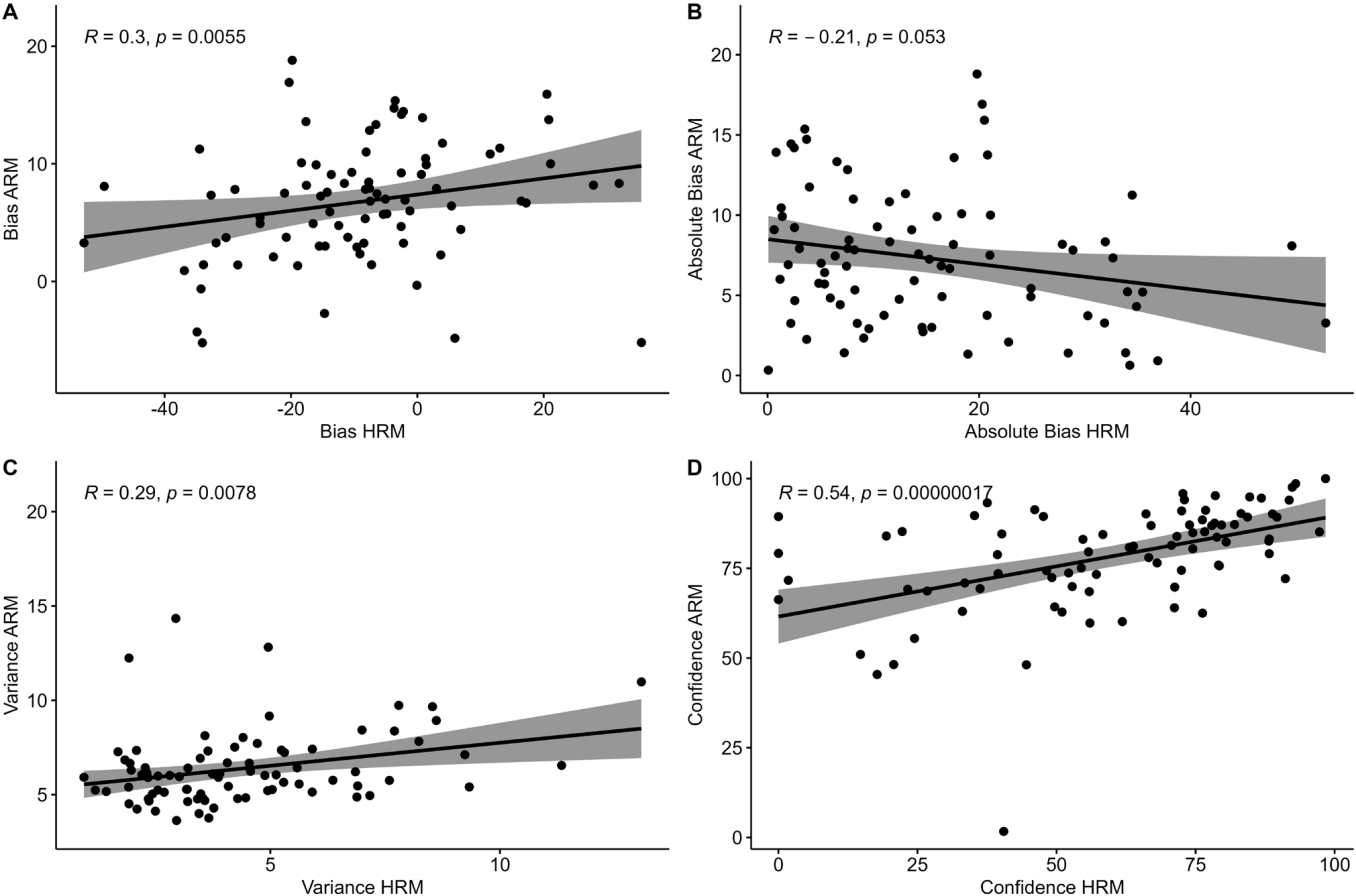
Associations between ARM and HRM variables. (A) Correlation between bias values. (C) Correlation between Absolute Bias values. (C) Correlation between Variance values. (D) Correlation between Confidence values. Statistic is Spearman Correlation co‐efficient. ARM, Audio Rate Matching; HRM, Heart Rate Matching.

To test discriminant validity of the HRM, we examined whether the same parameters from the HRM and the ARM correlated together (Figure 8). There were significant positive correlations between bias scores (*ρ* = 0.30, *p* = 0.006), between confidence ratings (*ρ* = 0.54, *p* < 0.001), and between variance scores (*ρ* = 0.29, *p* = 0.008), but not between absolute bias (*ρ* = −0.21, *p* = 0.053). These significant correlations suggest that the two tasks may share at least some common mechanisms, namely mechanisms of multisensory processing and integration.

**FIGURE 9:**
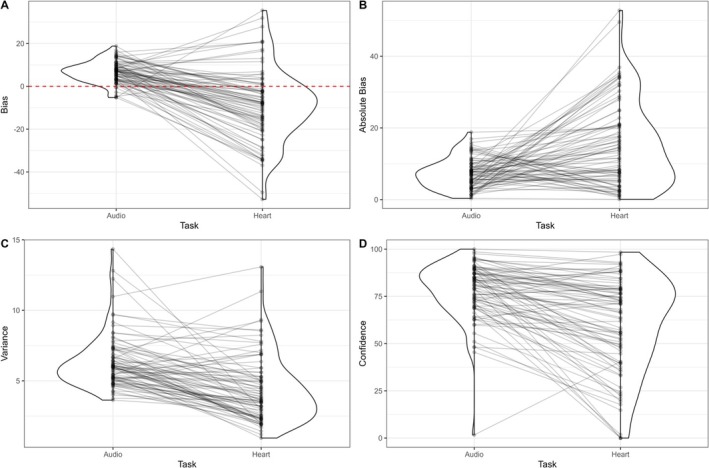
Distributions and differences between HRM and ARM variables. Individual lines connect data from the same participant. Graphs compare ARM and HRM performance in terms of (A) Bias values, (B) Absolute Bias, (C) variance, (D) confidence. ARM, Audio Rate Matching; HRM, Heart Rate Matching.

Next, we examined differences between the ARM and the HRM (Figure 9). Bias was significantly lower in the heart task, *t*(82) = 8.74, *p* < 0.001, 95% CI [12.29, 19.55]; absolute bias was higher in the heart task, *t*(82) = −5.30, *p* < 0.001, 95% CI [−10.79, −4.90]; variance was higher in the audio task, *t*(82) = 7.12, *p* < 0.001, 95% CI [1.45, 2.58]; and confidence was higher in the audio task, *t*(82) = 7.51, *p* < 0.001, 95% CI [13.62, 23.44]. These results suggest key differences in how people estimate audio rate compared to heart rate, with estimations of auditory rate tending to be more accurate, yet also more variable.

### 6.7 HRM and Interoceptive Questionnaires

Full correlation matrices between Heart Rate Matching and the questionnaires are reported in Supporting Information.

Neither Bias nor absolute Bias on the HRM significantly correlated with total scores or any subscales of the MAIA‐2, the IAS, the IATS.

Variance on the HRM was significantly negatively correlated with the emotional awareness subscale of the MAIA‐2 (*ρ* = −0.25, *p* = 0.01), but not with any other subscale. This correlation was not significantly different from the correlation between ARM variance and the emotional awareness subscale of the MAIA‐2 (*z* = −1.79, *p* = 0.073, 95% CI [−0.475, 0.022]).

Confidence on the HRM was significantly positively correlated with attention regulation (*ρ* = 0.29, *p* = 0.004), body listening (*ρ* = 0.20, *p* = 0.048), and body trust (*ρ* = 0.28, *p* = 0.006) subscales of the MAIA‐2, as well as the total MAIA score (*ρ* = 0.29, *p* = 0.004). It also significantly correlated with the IAS total score (*ρ* = 0.30, *p* = 0.003). None of these correlations were significantly different from the equivalent correlation with ARM confidence, for attention regulation (*z* = 1.21, *p* = 0.228, 95% CI [−0.085, 0.35]), body listening (*z* = 1.554, *p* = 0.12, 95% CI [−0.046, 0.392]), body trust (*z* = 1.73, *p* = 0.08, 95% CI [−0.027, 0.41]), MAIA‐2 total score (*z* = 1.286, *p* = 0.198, 95% CI [−0.076, 0.36]), or IAS total score (*z* = 0.094, *p* = 0.93, 95% CI [−0.21, 0.23]). These findings suggest the relationship between interoceptive questionnaires and confidence on the HRM was driven by a more general process than specific confidence in interoceptive judgments.

### 6.8 HRM, Dissociation, Alexithymia, and Anxiety

Neither Bias, absolute Bias, nor Variance on the HRM or ARM were significantly correlated with total scores or the subscales of the CDS, TAS, or STICSA questionnaires (see Supporting Information).

Confidence on the HRM was significantly negatively correlated with total score on the TAS (*ρ* = −0.24, *p* = 0.02), as well as the difficulty identifying feelings subscale (*ρ* = −0.26, *p* = 0.01), while confidence on the ARM was significantly negatively related to the difficulty describing feelings subscale (*ρ* = −0.19, *p* = 0.03). Neither of the correlations with HRM confidence was significantly different from the equivalent correlation with ARM confidence for the total score of the TAS (*z* = −0.62, *p* = 0.54, 95% CI [−0.293, 0.153]) or the difficulty identifying feelings subscale (*z* = −0.448, *p* = 0.654, 95% CI [−0.268, 0.167]).

### 6.9 Internal Reliability

Since half of trials begin with a “low” starting BPM and half begin with a “high” starting BPM, and we know this can produce different values of bias, we examined correlations between the first and last three trials, separately for “low” and “high” starting trials. In practice, we calculated Bias, Absolute Bias, Variance and Confidence for the first and last three trials of “low” and “high” starting trials, and then correlated the outcomes from the first and last trials.

Correlation coefficients and significance values are reported in Table 2. Across both HRM and ARM tasks, variance shows extremely poor internal consistency, suggesting that it is not a reliable outcome measure. Bias, absolute bias, and confidence ratings do have strong internal consistency for both the HRM and the ARM tasks.

**TABLE 2:** Split‐half reliability means and correlation tests for HRM and ARM outcome measures.

HRM outcome	Starting condition					
	Low			High		
	First	Last	Correlation	First	Last	Correlation
Bias	−17.32	−14.56	*r* = 0.81 *p* < 0.001	0.78	−5.62	*r* = 0.78 *p* < 0.001
Abs Bias	20.69	19.94	*r* = 0.73 *p* < 0.001	14.88	17.22	*r* = 0.59 *p* < 0.001
Variance	4.15	3.95	*r* = −0.11 *p* = 0.42	5.46	4.44	*r* = 0.07 *p* = 0.6
Confidence	59.85	63.72	*r* = 0.92 *p* < 0.001	56.88	61.78	*r* = 0.90 *p* < 0.001

ARM outcome	Starting condition					
	Low			High		
	First	Last	Correlation	First	Last	Correlation
Bias	−0.97	1.97	*r* = 0.61 *p* < 0.001	14.2	11.5	*r* = 0.66 *p* < 0.001
Abs Bias	8.14	7.64	*r* = 0.47 *p* < 0.001	15.3	12.9	*r* = 0.75 *p* < 0.001
Variance	9.07	9.89	*r* = 0.04 *p* = 0.69	10.4	10.4	*r* = 0.13 *p* = 0.19
Confidence	77.8	78.7	*r* = 0.73 *p* < 0.001	77.8	78.4	*r* = 0.77 *p* < 0.001

## 7 Discussion—Experiment 2

The second experiment demonstrated that the HRM task does significantly correlate with a Heartbeat Counting task if participants are not instructed to only report explicitly counted heartbeats. These instructions reflect how participants were classically instructed to complete the task (Schandry 1981), though researchers advocate adapted instructions to ensure it more accurately measures the construct of accuracy in detecting individual heartbeats (Desmedt et al. 2018). The HRM task appears to be more closely associated with the original Heartbeat Counting task rather than the adapted Heartbeat Counting task, though this correlation, while significant, was between a small and medium correlation (*r* = 0.26), lower than might be expected to provide firm evidence for convergent validity. This suggests that the HRM task is more likely to be a measure of how well an individual can estimate their heart rate over a period of time, rather than a measure of how well they can perceive individual heartbeats. To clarify this interpretation of the HRM task, Experiment 3 tested the association between the HRM and the Heart Rate Discrimination (HRD) task, which is designed to assess the ability to estimate heart rate (Legrand et al. 2022).

In addition, Experiment 2 found associations between the HRM and a matched exteroceptive task, the ARM task, suggesting that the HRM may not be a pure measure of interoceptive ability because it likely involves some multi‐sensory binding between interoceptive and exteroceptive senses.

The experiment also found limited correlations between bias scores on the HRM and both questionnaires designed to assess interoception or questionnaires assessing constructs related to interoception such as anxiety, alexithymia, and dissociation. We did observe several correlations between questionnaire measures and average confidence on the HRM, suggesting a close association between self‐reported confidence in monitoring heart rate in a behavioral task and self‐reported interoceptive ability on trait questionnaires, both of which have previously been subsumed under the construct of Interoceptive Sensibility (Garfinkel et al. 2015). However, z‐tests found that these correlations were not significantly different from matched correlations to the exteroceptive control task, suggesting the underlying relationship may be due to more general mechanisms not exclusive to interoception.

Finally, we demonstrated excellent internal reliability of Bias, absolute Bias, and confidence on the HRM and ARM tasks, but poor internal reliability of the Variance metric. This is similar to the poor test–retest reliability reported for the Slope metric of the HRD task (Legrand et al. 2022), which was also purported to measure consistency of heart rate estimations. Test–retest reliability of the Variance metric will also be tested in Experiment 3.

In Experiment 3, we sought to clarify the relationship between the HRM and the HRD task. We also assessed the test–retest reliability of the HRM and whether it was a more engaging task for participants compared to the HRD.

## 8 Methods—Experiment 3

### 8.1 Participants

A power analysis was conducted to determine an ideal sample size for a one‐tailed correlation test with a large effect size (*r* = 0.5), an alpha level of 0.05% and 95% power. The estimated correlation was chosen because tests of test–retest reliability have found similar sized correlations for similar tasks (Legrand et al. 2022), and we expected both convergent validity and test–retest reliability to be high.

The power analysis recommended a sample size of 34 participants. Thirty‐five participants were recruited in total, with 1 participant only attending the first session; therefore, 34 participants completed the full study.

### 8.2 Design

Participants completed two sessions, involving a combination of behavioral tasks, questionnaires, and physiological recordings. Where possible, participants completed the sessions 7 days apart, at the same time of day.

### 8.3 Resting Heart Rate

Participants were asked to remain still and silent for 5 min while heart rate was recorded. Heart rate across all tasks was recorded with electrocardiography, with disposable electrodes attached at both shoulders and the lower back (the same setup is described in Experiment 1).

### 8.4 Heart Rate Matching

The HRM task was implemented in Gorilla with eight trials. Following inspection of the previous experiments, we widened the minimum and maximum values of response to 10 BPM and 150 BPM to allow for a wider range of heart rate beliefs. In addition, “low” trials started at a BPM between 20 and 30 BPM, while “high” trials started between 130 and 140 BPM. No participants had an average resting heart rate outside of these bounds. The step‐change in reported BPM with each key press was changed to 1 BPM from 2 BPM, to allow participants more precision in their responses. Finally, the duration of trials were unfixed, meaning participants could adjust their response as much as they wished before submission. Unfixed durations are the standard approach for Method of Adjustment approaches (Gescheider 2013), but were not possible to implement in the previous two experiments because they estimated heart rate with rPPG methods which require a video recording of greater than 30 s to reliably estimate heart rate (Di Lernia et al. 2024). There was a maximum duration of 2 min for each trial, and participants saw a warning message 20 s prior to this maximum time to ensure they submitted their response.

### 8.5 Heart Rate Discrimination (HRD; Legrand et al. 2022)

The HRD was conducted in Psychopy. The HRD is a two‐interval forced choice task. On each trial, the participant was prompted to listen to their own heartbeat for 5 s. Then, a set of auditory tones was played through the computer speakers. The frequency of the auditory tones was determined with an adaptive staircase procedure. Participants then judged whether the auditory tones were faster or slower than their heart rate. Overall, there were 60 trials, with 12 trials included as “catch” trials and 48 trials as “psi” trials.

### 8.6 Dundee Stress State Questionnaire, Short Version (DSSQ)

The short version of the DSSQ (Matthews et al. 2013) is a 21‐item questionnaire, with items divided into three 7‐item subscales of Task Engagement, Distress, and Worry. Each item is a statement (see Table 3 for example items), and participants are asked to rate how much they agree with that statement on a 5‐point Likert scale. The DSSQ was administered before and after the HRM in the first session, and before and after the HRD in the second session. In the “pre‐DSSQ”, questions are framed to ask participants to answer how they are feeling right now, whereas the “post‐DSSQ” questions ask participants in relation to how they felt during the task.

**TABLE 3:** Example items of the DSSQ subscales, short form.

DSSQ subscale	Item
Task engagement	My attention was directed toward the task
Distress	I felt tense
Worry	I felt concerned about the impression I was making

### 8.7 Procedure

The study consisted of two sessions. In the first session, after providing informed consent, a resting heart rate scan was conducted. Following the scan, participants completed the HRM task, with the DSSQ completed before and after the task. The second session also began with the resting heart rate scan, followed by the HRM task. After this task, participants completed the HRD task, with the DSSQ completed before and after the task.

### 8.8 Analysis

To explore the test–retest reliability of the HRM, Intra‐Class Coefficients (ICCs) and their 95% confidence intervals were calculated for the HRM outcome measures of bias, variance, and confidence. ICC was calculated as a two‐way mixed effects model based on the mean of multiple measurements (Koo and Li 2016).

To explore the convergent validity of the HRM with the HRD, correlations were calculated between HRM outcome variables at the second session and HRD outcome variables.

To explore differences in stress changes following the HRM and HRD, we compared DSSQ subscale mean scores (Task Engagement, Distress and Worry) between the two administrations of the questionnaire with linear mixed models separately, for each of the subscales. For each model, time (pre‐task, post‐task) and task (HRM, HRD) were included as fixed effects, as well as the interaction between the two. A by‐subject random intercept was included in each model to account for the repeated‐measures design.

## 9 Results—Experiment 3

The 34 participants had a mean age of 20.4 (SD = 3.33), and 29 identified as Female (85.2%), 5 identified as Male (14.8%).

### 9.1 Test–Retest Reliability of the HRM

Most HRM outcome measures were normally distributed according to Shapiro–wilk tests in both session 1 and session 2. Absolute bias was non‐normally distributed for both sessions, and the variance was non‐normally distributed for session 2. No participants were excluded on the basis of examination of reaction times or key presses. Responses were inspected to determine if they fell outside of 2.5 times the Median Absolute Deviation from the median value, but no participants were excluded on this basis (Leys et al. 2013).

**FIGURE 10:**
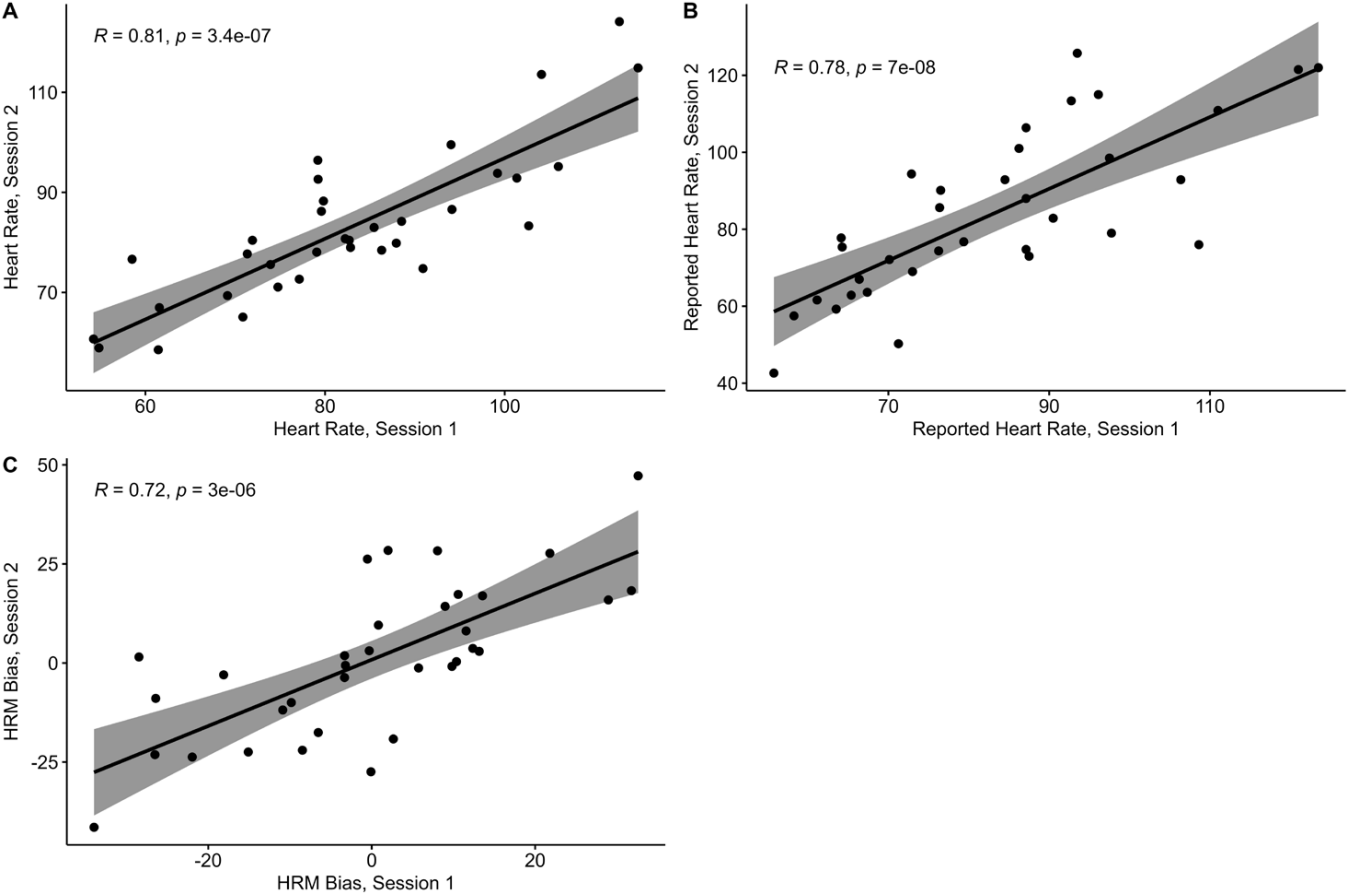
Correlations between HRM variables assessed at two sessions. Statistics are Spearman correlation coefficients. HRM, Heart Rate Matching.

A set of paired *t*‐tests found no significant differences between the two sessions for each outcome variable of the HRM (Table 4), apart from variance which was significantly higher in session 1 (*t*(33) = 2.89, *p* = 0.006). Inspection of ICC values for the outcome variables revealed good test–retest reliability (0.75 < ICC < 0.9) for heart rate and estimated heart rate, according to widely accepted guidelines for interpreting ICC values (Koo and Li 2016). Bias, Variance and Confidence all showed moderate test–retest reliability (0.5 < ICC < 0.75), while absolute Bias demonstrated poor test–retest reliability (ICC < 0.5). Most HRM task metrics therefore have moderate or good test–retest reliability, which was also confirmed by Spearman correlations (Figure 10). The HRM took on average 6.59 min to complete (SD = 2.25).

**TABLE 4:** Comparison of Heart Rate Matching performance between sessions.

Variable	Session 1 mean	Session 2 mean	Paired *t*‐tests	ICC [95% CI]
Heart rate (BPM)	82.72	82.92	*t*(33) = −0.13, *p* = 0.90	0.844 [0.71, 0.92]
Reported heart rate (BPM)	82.94	83.94	*t*(33) = −0.42, *p* = 0.67	0.763 [0.58, 0.87]
Bias	0.23	1.02	*t*(33) = −0.34, *p* = 0.74	0.722 [0.51, 0.85]
Absolute bias	12.99	14.96	*t*(33) = −0.89, *p* = 0.38	0.343 [0.01, 0.61]
Variance	8.58	7.07	*t*(33) = 2.89, *p* = 0.006	0.719 [0.46, 0.86]
Confidence	54.76	56.4	*t*(33) = −0.84, *p* = 0.41	0.627 [0.37, 0.79]

### 9.2 Heart Rate Discrimination Performance

For the HRD, data from one participant was excluded as their threshold exceeded 2.5 times the median absolute deviation of threshold (Leys et al. 2013). An additional two participants were excluded because of a mismatch between the ECG assessment of heart rate and the heart rate recorded by the task itself. The HRD was designed to only allow heart rates between 50 and 120 BPM; if a heart rate was outside of this range, the task displayed a message instructing the participant to stop moving and would only move onto the next trial once it detected that heart rate had fallen into the acceptable range. These two participants often had a heart rate greater than 120 BPM, meaning this error occurred regularly while they completed the task. Since the HRD relies upon accurate input of heart rate to correctly determine stimulus values, these participants were excluded from analyses of the HRD because we cannot be sure that their performance estimates were accurate. Therefore, the following analyses involving the HRD are with *N* = 31 participants.

Threshold and Slope values were both normally distributed. On average, participants had a threshold of −5.45, and a slope of 10.37. These values closely match previously reported values for these parameters (Legrand et al. 2022), which report threshold in a first and second session of −6.97 and −8.5, respectively, and slopes of 15.34 and 11.96, respectively.

### 9.3 Associations Between Heart Rate Matching and Heart Rate Discrimination Tasks

**FIGURE 11:**
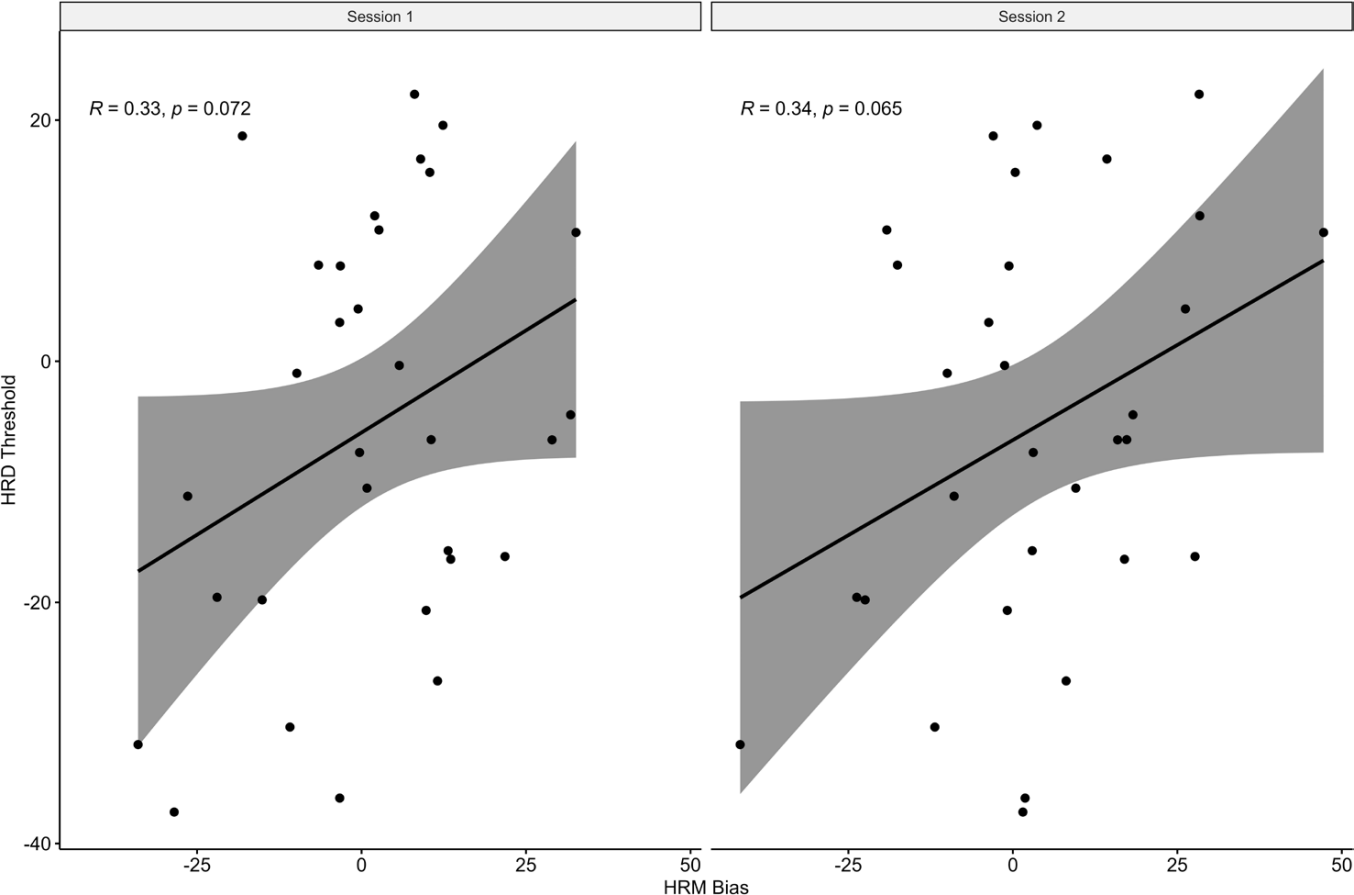
Correlation between Heart Rate Discrimination Threshold and Heart Rate Matching Bias. HRD, Heart Rate Discrimination; HRM, Heart Rate Matching.

**FIGURE 12:**
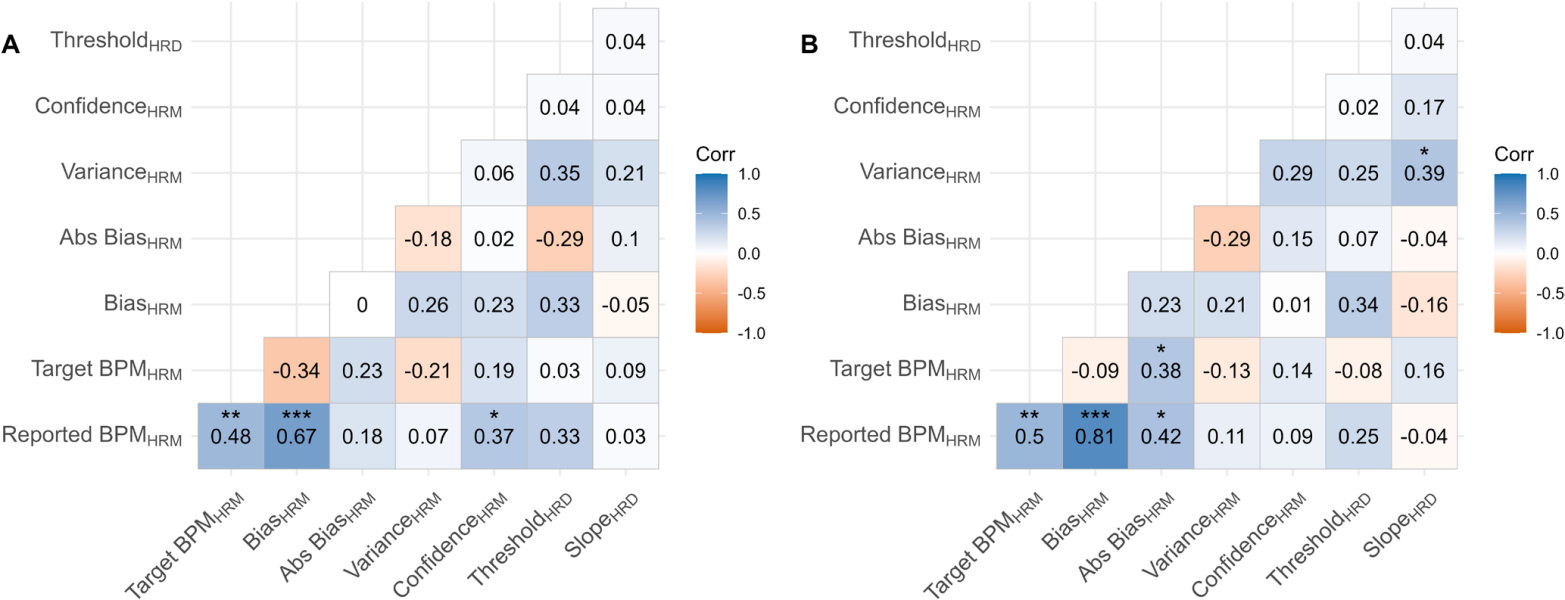
Associations between Heart Rate Matching and Heart Rate Discrimination variables. (A) HRM values assessed at Session 1. (B) HRM values derived from Session 2. BPM, beats per minute; HRD, Heart Rate Discrimination; HRM, Heart Rate Matching. *p < 0.05, **p < 0.01, ***p < 0.001.

There were moderate but nonsignificant Pearson correlations between bias and threshold, for both Bias estimated from the HRM at session 1, *r* = 0.33, 95% CI [−0.03, 0.61], *t*(29) = 1.86, *p* = 0.072, and at session 2, *r* = 0.34, 95% CI [−0.02, 0.62], *t*(29) = 1.92, *p* = 0.065 (Figure 11). The effect size greater than *r* = 0.3 for both correlations suggests that threshold derived from the HRD and bias derived from the HRM were moderately correlated. The only significant correlation between the HRM and HRD variables was between slope and the variance of bias in session 2 (Figure 12), though this same correlation was not significant in session 1.

In addition, threshold had a low, nonsignificant correlation with average heart rate in session 1, *r* = 0.03, 95% CI [−0.33, 0.38], *t*(29) = 0.16, *p* = 0.877, and Session 2, *r* = −0.08, 95% CI [−0.42, 0.28], *t*(29) = −0.42, *p* = 0.675. Threshold was also nonsignificantly correlated with reported BPM at both Session 1, *r* = 0.33, 95% CI [−0.03, 0.61], *t*(29) = 1.87, *p* = 0.071 and Session 2, *r* = 0.25, 95% CI [−0.33, 0.38], *t*(29) = 1.36, *p* = 0.183.

### 9.4 Task‐Related Engagement, Distress, and Worry

Reliability of the DSSQ subscales was acceptable according to Cronbach's alpha (> 0.70) for the engagement subscale at the post‐task measurement (pre‐task: 0.69, post‐task: 0.8), the distress subscale at both timepoints (pre‐task: 0.79, post‐task: 0.90) and the worry subscale at post‐task (0.70) but not pre‐task (0.61).

For task engagement, no significant fixed effects were found for either time (*B* = 0.57, *t*(100.1) = 0.87, *p* = 0.385), task (*B* = −0.38, *t*(100.35) = −0.57, *p* = 0.57) or the interaction (*B* = −1.13, *t*(100.1) = −1.21, *p* = 0.23), suggesting engagement did not significantly change from pre‐task to post‐task for either the HRM or the HRD.

For distress, a significant effect of time was found (*B* = 3.23, *t*(100.2) = 3.29, *p* = 0.001) suggesting that task‐related distress was higher at the post‐task assessment for both the HRM and HRD compared to the pre‐task assessment. Neither the effect of task (*B* = 0.76, *t*(100.5) = 0.77, *p* = 0.44) nor the interaction (*B* = 0.8, *t*(100.16) = 0.57, *p* = 0.57) was significant.

**FIGURE 13:**
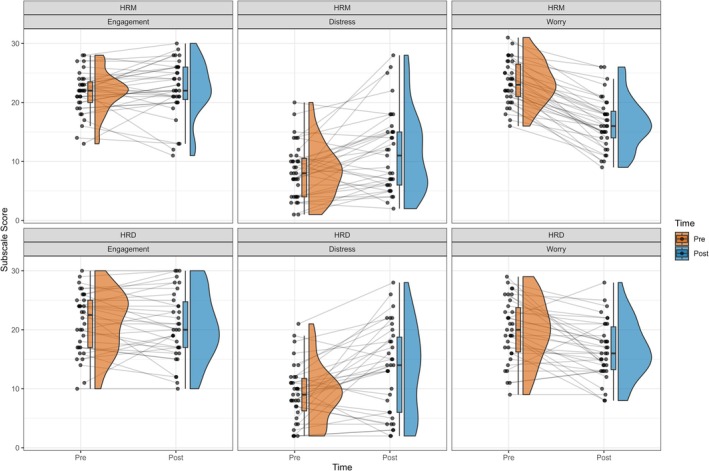
Task Engagement, Distress, and Worry in response to the Heart Rate Matching and Heart Rate Discrimination tasks. Individual lines connect data from the same participant. HRD, Heart Rate Discrimination; HRM, Heart Rate Matching.

For worry, there were significant effects of both time (*B* = −6.91, *t*(100.2) = −9.89, *p* < 0.001) and task (*B* = −3.4, *t*(100.5) = −4.93, *p* < 0.001), as well as a significant interaction between the two (*B* = 3.62, *t*(100.2) = 3.64, *p* < 0.001). On inspection of the data (Figure 13), the HRM task appeared to decrease levels of worry from pre‐task to post‐task. However, the pre‐task level of distress was higher for the HRM compared to the HRD, whereas the post‐task level of worry was similar for the two tasks. This suggests that the reduction in worry caused by the HRM may simply be an artifact of the higher worry participants had prior to the start of the HRM compared to the HRD, which was assessed at the beginning of the first session. An alternative interpretation is that the pre‐task DSSQ assessment for the HRD was conducted after the HRM in the second session, meaning that worry may already have been reduced to a level that was low enough to be subsequently unaffected by the HRD task.

## 10 Experiment 3—Discussion

Experiment 3 investigated the test–retest reliability of the HRM task and examined associations between metrics of the HRM and HRD tasks as well as their impact on task engagement.

First, we found evidence of moderate‐to‐good test–retest reliability for the main outcomes of the HRM task. This suggests the task itself has good reliability, particularly combined with Experiment 2 demonstrating good internal reliability of the task.

Second, we found moderate correlations between the primary outcome of the HRM task, bias, and the primary outcome of the HRD task, threshold. Both variables are thought to capture how closely an individual's estimate of their heart rate deviates from their actual heart rate. Despite the association, there were differences between the tasks in the average bias and threshold, with the HRM suggesting that people slightly over‐estimated their heart rate by 0.23 BPM (Session 1) and 1.02 BPM (Session 2) on average. The HRD, in contrast, found that people under‐estimated their heart rate by 5.45 BPM on average.

Finally, there appeared to be no difference between the HRM and the HRD in their effects on task‐related engagement. Advocates of Method of Adjustment tasks, such as the HRM, suggest that they are more engaging for participants than alternative forced choice tasks, such as the HRD. This has rarely been empirically tested; however, our results suggest that matching tasks are no more engaging than forced choice tasks. Task‐related distress was increased by both tasks. Task‐related worry was reduced by only the HRM, although this could be due to a higher pre‐task level of worry for the HRM compared to the HRD, as post‐task worry was at a similar level for both the HRM and HRD.

## 11 General Discussion

Across three experiments, with three independent samples, the present study aimed to verify the construct validity of a novel task designed to assess the ability to monitor one's own heart rate. This involved testing its convergent and discriminant validity, and its internal and test–retest reliability.

### 11.1 Convergent Validity of the HRM

In all three experiments, we examined the association between the Heart Rate Matching task and other tasks designed to measure the ability to either perceive one's own heartbeat or estimate heart rate. In Experiment 1, we found no significant correlations between HRM bias or variance with scores from a 6‐AFC Heartbeat Discrimination task or the Heartbeat Counting task with adapted instructions to only count explicitly felt heartbeats (Desmedt et al. 2018), though there was a correlation between confidence ratings on both tasks. This suggests low convergent validity of the HRM with tasks designed to assess the ability to perceive heartbeats. In Experiment 2, we found a significant positive correlation between bias on the HRM and Heartbeat Counting score using the original instructions of the task (Schandry 1981). In Experiment 3, we found a medium‐sized correlation (Cohen 1988), though nonsignificant, between bias on the HRM and bias on the HRD task.

We speculate that the difference in correlations between different versions of the Heartbeat Counting task may have been caused by a key difference in the tasks between Experiment 1 and 2. In the first experiment, participants were asked to only count heartbeats that they could explicitly feel (Desmedt et al. 2018). In the second experiment, no such instructions were provided, with the task resembling the original task (Schandry 1981). The adapted instructions are intended to minimize the influence of non‐interoceptive processes on performance, such as one's prior knowledge of heart rate or time estimation. It could be argued that the different instructions result in two distinct tasks which measure different processes. The original Schandry task, while intended to assess objective accuracy at detecting heartbeats, may be better interpreted as a measure of the ability to estimate heart rate because it can be influenced by both sensory perception of each heartbeat and by one's prior knowledge of heart rate. In contrast, the adapted task is a more stringent measure of someone's ability to count each individual heartbeat and therefore better captures the concept of objective accuracy at detecting heartbeats (Garfinkel et al. 2015). Our findings that bias on the HRM significantly correlated with the original, but not the adapted, version of the Heartbeat Counting task suggest that the HRM task should not be interpreted as an interoceptive accuracy task, but instead a task which measures beliefs about heart rate. This position is further supported by findings from Experiment 3 of a moderate but nonsignificant association between the HRM and the HRD task. However, a comparison of results across the three experiments using null hypothesis significance testing (see Supporting Information) suggests that the lack of significant correlation with the adapted Heartbeat Counting task may be due to a lack of power in Experiment 1, and future studies will need to verify if this is the case.

In Experiment 2, we also tested convergent validity of the HRM with three different self‐report questionnaires designed to assess interoceptive ability, the Interoceptive Accuracy Scale (IAS), the Interoceptive Attention Scale (IATS) and the Multi‐Dimensional Assessment of Interoceptive Awareness (MAIA). The IAS and the MAIA scores were significantly positively correlated with confidence ratings on the HRM, suggesting that self‐reported interoceptive accuracy and awareness aligned with confidence ratings on the task. Examining the subscales of the MAIA, we found that the body trust subscale was correlated with both bias and confidence scores on the HRM, and the attention regulation subscale was significantly positively correlated with confidence scores. State confidence ratings on interoceptive tasks and trait scores on self‐report interoceptive questionnaires often show close associations, and have previously been grouped together as a single factor such as Interoceptive Sensibility (Garfinkel et al. 2015). However, the relations between HRM confidence and the interoceptive questionnaires were found to be statistically similar to the association between ARM confidence and interoception questionnaires, so we are unable to conclude that these relations are based on purely interoceptive mechanisms.

Interestingly, we found no significant correlations between the original Heartbeat Counting task and any of the three interoceptive questionnaires. In past research with the IAS, there was a significant positive correlation between the adapted Heartbeat Counting task and IAS across two samples (Murphy et al. 2020), although others have also found nonsignificant correlations between the IAS and cardiac interoceptive accuracy (Brand et al. 2023; Folz et al. 2024). In our experiment, the nonsignificant result may be due to the instructions provided in Experiment 2 for Heartbeat Counting, which used the classic instructions, whereas previous research comparing IAS and Heartbeat Counting used the adapted instructions. However, given null findings in other publications, it is also possible that the relationship between IAS and cardiac interoceptive accuracy is small.

### 11.2 Discriminant Validity of the HRM

We found significant positive correlations between the HRM and an exteroceptive control task, the Audio Rate Matching (ARM) task. The ARM was designed to mirror the HRM but instead required estimating the rate of an exteroceptive auditory tone instead of one's own heart rate. Each outcome variable of the two tasks, bias, variance, and confidence, was all significantly positively correlated between the two tasks. This suggests that the two tasks engage multisensory integration processes, implying that the HRM does not rely on exclusively interoceptive processes. For example, previous studies have indicated that judging simultaneity between audio and visual stimuli explains around a third of variance in performance on an interoceptive accuracy task (Knapp et al. 1997), and may play a similar role with the HRM and ARM tasks.

Although the ARM and the HRM are designed to be highly similar tasks, the nature of the signal that participants are attempting to detect in each task means that different processes are likely involved. The ARM presents a suprathreshold, easily detectable and reliably occurring auditory tone. The task to match the visual stimulus to the auditory stimulus is a task of exteroceptive multi‐sensory integration—of listening to the auditory tone, watching the visual pulse, and taking steps to match the two together. In contrast, the heartbeat is ephemeral, fading in and out of detectability and changing in response to the external environment. For some people and for some trials, where the heartbeat becomes a suprathreshold stimulus, the heartbeat may closely resemble the auditory tone in the ARM. In this case, performance on the HRM is likely to reflect a similar multi‐sensory integration process, albeit between exteroceptive and interoceptive stimuli. However, for other trials where the heartbeat is less perceptible, the task becomes more of a guessing game. Without a consistently clear stimulus to match to, the estimate of heart rate will be based on beliefs and knowledge about the heart as well as the few heartbeats that can be detected. It is likely for this reason that we found a significantly greater under‐estimation for the HRM compared to the ARM. However, the finding of close correlations between ARM and HRM variables suggests that non‐interoceptive processes such as multi‐sensory integration are likely to be involved in both tasks.

### 11.3 Reliability of the HRM

For both the HRM and the ARM, there were strong positive correlations between bias and confidence outcomes that were calculated with different subsets of trials. Bias and confidence therefore have good internal consistency reliability, meaning that the task appears to be measuring a similar construct from trial to trial. In contrast, variance had poor internal reliability. Variance is designed to capture the consistency of the bias value from trial to trial and can be thought of as the inverse of precision, or the consistency of the heart rate estimate. It should be noted that a similar measure of precision from the HRD task also showed poor test–retest reliability (Legrand et al. 2022), implying that it might be particularly challenging to reliably measure uncertainty of heart rate beliefs. Experiment 3 demonstrated moderate to good test–retest reliability of bias, variance, and confidence metrics of the HRM. Interoceptive ability (whether accuracy or beliefs) is likely to be state‐dependent, and therefore future research should look to estimate test–retest reliability of the HRM across different states (e.g., pre‐ and post‐stress) as well as different occasions (e.g., Wittkamp et al. 2018).

### 11.4 Task Burden

Experiment 3 revealed that the HRM and HRD have a similar level of burden for the participant, having similar levels of engagement as well as task‐related distress and worry. Since the HRM is noticeably quicker to administer, ranging from 6 to 12 min, and can be administered online, we suggest a combination of these two tasks may be useful in assessing heart rate estimation ability. Indeed, the adaptive psychophysical procedure of the HRD means that if researchers have a good estimate of an individual's heart rate estimation ability prior to administering the task, then the number of trials can be reduced. Researchers could therefore ask participants to complete the HRM remotely first and use the bias estimate to ensure a shortened version of the HRD task can be completed in the lab. Our study suggests that if testing in a dedicated lab space is not an option, if specialized equipment to measure cardiac timing is not available, or if a task of shorter duration is required, researchers may consider using the HRM task over the HRD task to assess heart rate beliefs.

### 11.5 rPPG Limitations

The HRM task was designed to measure interoception quickly, and at scale, by offering an online task that can be paired with rPPG methodologies to estimate heart rate remotely (Di Lernia et al. 2024). However, a severe limitation of rPPG is its poor performance in accurately measuring heart rate in people with darker skin tones (Nowara et al. 2020). As a result, we included only white participants in our Experiment 2, in which rPPG was our only measure of heart rate. This greatly limits the generalizability of findings from this experiment and undercuts the goal of providing a solution to measure interoception in larger samples. rPPG technologies are continually being developed to reduce this measurement bias (e.g., Chari et al. 2020). The same limitation does not apply in Experiments 1 and 3, where heart rate was assessed with ECG. In addition, we experienced a large data loss due to poor quality of webcam video recordings. After filtering for this and careless responding (e.g., too fast responses), in the HRM only 94 of 149 participants remained, a loss of just over a third of participants, which is similar to previous studies using rPPG in online studies (Di Lernia et al. 2024). To some extent, poorer data quality is the trade‐off for collecting large samples in online studies, but efforts should be made to minimize data loss. Researchers may need to take steps such as screening for level of webcam quality, before recruiting participants to take part. Given these limitations, researchers might consider recording heart rate in the HRM with alternative methods such as finger pulse PPG via smartphone cameras, which is incorporated in the Phase Adjustment Task (Plans et al. 2021) although webcam‐based rPPG may be preferable in some situations (see Pirzada et al. 2023 for a review). An additional limitation of our samples in Experiments 1 and 3 were the large prevalence of female participants, which further limits generalizability of these findings to other demographics.

### 11.6 How Does the HRM Fit Into Wider Interoception Frameworks?

A recent paper has proposed a novel framework for defining and conceptualizing the measurement of interoception (Desmedt, Luminet, Maurage, et al. Desmedt, Luminet, Maurage, and Corneille 2023). Interoception is divided into broad categories of interoceptive attention, interoceptive sensing, interoceptive interpretation and interoceptive memory, each of which can include both conscious and nonconscious processes. Each of these categories can further be underpinned by more specific constructs—for example, they suggest that interoceptive sensing is underpinned by interoceptive detection (accuracy), magnitude and localization. Measures can then be directly tied to each of these specific constructs—for example, the original heartbeat counting task is defined as a “measure of the capacity to estimate heart rate via mental counting”. Their final recommendation is to specify both bodily domain (e.g., heart, respiration, gastro‐intestinal) and measurement type (e.g., self‐report, objective) as past research has demonstrated minimal convergence between interoception of different bodily domains and measurement types. Within this framework, we believe that the HRM task would be considered a cardiac interoception task within the category of interoception detection as “a measure of the capacity to estimate heart rate via matching”. This delineates the HRM from tasks designed to measure the ability of an individual to perceive each heartbeat, such as the Heartbeat Discrimination task or the Heartbeat Counting task with adapted instructions to only count heartbeats explicitly felt. Instead, the HRM would align more with measures designed to estimate ability to detect heart rate, such as the HRD task (Legrand et al. 2022) or the original heartbeat counting task (Schandry 1981), which is supported by our findings which suggest their convergent validity with the HRM, though further confirmatory investigations with appropriately powered samples are required to confirm this interpretation.

Crucially, Desmedt and colleagues place a key emphasis on ensuring that newly developed tasks have good psychometric properties according to the principles of construct validity (Strauss and Smith 2009). Any new task must be able to demonstrate good convergent and discriminant validity, as well as high internal and test–retest reliability. In this study we demonstrated some evidence that the HRM task converges with the original Heartbeat Counting task and the HRD tasks, but does not converge with the adapted Heartbeat Counting task and the Heartbeat Discrimination task. There was weak evidence for discriminant validity of the HRM task, given that performance correlated with an ARM task. Internal reliability was good for the bias and confidence variables, but not for variance. Lastly, test–retest reliability was moderate‐to‐good for variables derived from the HRM task. Establishing construct validity for a new measure is a continuous requirement for any measure and we implore future users of the HRM to fully report available reliability and validity metrics of the task.

A remaining issue with the HRM and other tasks assessing ability to estimate heart rate, are the different sources of information that can be used to estimate heart rate. An estimate of heart rate can be influenced by bottom‐up sensory information, the felt heartbeats, but can also be influenced by prior knowledge, experiences or held beliefs about the heart rate. In terms of construct validity, this is a problem of construct representation (Whitely 1983). Construct representation refers to the understanding of how a psychological process leads to a given response on a trial. We have a theory that interoceptive sensing influences HRM performance, but it may be that other processes (e.g., knowledge of heart rate) can also influence HRM performance. A way to directly test the construct representation of the HRM is to systematically vary the bottom‐up sensory information and the top‐down prior beliefs and assess their respective and conjoint influences on HRM performance. Systematic variations of the incoming heartbeat signal can be achieved through non‐invasive perturbation techniques such as breath‐holding (Smith et al. 2021), while variations of prior knowledge of heart rate can be achieved with false feedback on heart rate to individuals prior to completion of the task. Future studies should systematically vary sensory information and prior beliefs to test the construct representation of the HRM task.

Future studies may also wish to examine the trajectories of performance on the HRM task. Each trial begins with the set heart rate being far from the average resting heart rate, and participants must gradually adjust its pace. Examining how quickly participants adjust the heart to match their own may present an implicit measure of interoceptive variables such as their confidence in their decision‐making, similarly to how subtle variations in finger movements during motor tasks provide online, implicit measures of the decision‐making process (Dotan et al. 2018). The ability to assess the online decision‐making process in the HRM is a potentially unique aspect of this task amongst other interoception tasks.

### 11.7 Conclusions

In conclusion, we tested the construct validity of a novel Heart Rate Matching task across three experiments. Bias, the extent to which an individual under‐ or over‐estimated their heart rate had excellent internal reliability and moderate‐to‐good test–retest reliability, and demonstrated convergent validity with other similar interoceptive tasks assessing estimation of heart rate (Legrand et al. 2022; Schandry 1981), but not tasks designed to assess ability to detect individual heartbeats (Brener et al. 1993; Desmedt et al. 2018). In addition, confidence ratings on the HRM correlated with other subjective self‐report assessments of interoceptive ability, though little evidence for discriminant validity was found. The HRM is freely available for researchers to use on Gorilla Open Materials (https://app.gorilla.sc/openmaterials/868003), and can be administered online in combination with rPPG methods to record true heart rate without the need for specialist equipment. Our findings suggest the HRM has acceptable convergent validity with tasks estimating interoceptive beliefs, as well as moderate test–retest reliability and good internal reliability, demonstrating its suitability for testing the ability to estimate one's own heart rate, an important aspect of cardiac interoception.

## Author Contributions

**Mariana Von Mohr:** conceptualization, methodology, data curation, formal analysis, writing – review and editing. **Manos Tsakiris:** conceptualization, supervision, resources, writing – review and editing, project administration, funding acquisition. **Jamie A. Moffatt:** conceptualization, methodology, software, data curation, investigation, validation, formal analysis, visualization, project administration, writing – original draft, writing – review and editing. **Markus R. Tünte:** formal analysis, visualization, writing – review and editing.

## Funding

This work was supported by H2020 European Research Council Consolidator (Grant ERC‐2016‐CoG‐724537) to M.T. under the FP7 for the INtheSELF project.

## Ethics Statement

Ethical approvals for all reported experiments were obtained from the University Research Ethics Committee at Royal Holloway, University of London prior to the onset of data collection.

## Conflicts of Interest

The authors declare no conflicts of interest.

## Supporting information


**Appendix S1:** psyp70284‐sup‐0001‐supinfo.docx.

## Data Availability

The data that support the findings of this study are openly available in Open Science Framework at https://osf.io/vgeq4/, reference number 10.17605/OSF.IO/VGEQ4.

## References

[psyp70284-bib-0001] Ainley, V. , M. Tsakiris , O. Pollatos , A. Schulz , and B. M. Herbert . 2020. “Comment on “Zamariola Et al. (2018), Interoceptive Accuracy Scores Are Problematic: Evidence From Simple Bivariate Correlations”—The Empirical Data Base, the Conceptual Reasoning and the Analysis Behind This Statement Are Misconceived and Do Not Support the Authors' Conclusions.” Biological Psychology 152: 107870. 10.1016/j.biopsycho.2020.107870. 32061687

[psyp70284-bib-0002] Bagby, R. M. , J. D. A. Parker , and G. J. Taylor . 1994. “The Twenty‐Item Toronto Alexithymia Scale—I. Item Selection and Cross‐Validation of the Factor Structure.” Journal of Psychosomatic Research 38, no. 1: 23–32. 10.1016/0022-3999(94)90005-1. 8126686

[psyp70284-bib-0003] Brand, S. , A. C. Meis , M. R. Tünte , et al. 2023. “A Multi‐Site German Validation of the Interoceptive Accuracy Scale and Its Relation to Psychopathological Symptom Burden.” Communications Psychology 1, no. 1: 1–13. 10.1038/s44271-023-00016-x. 39242870 PMC11332230

[psyp70284-bib-0004] Brener, J. , X. Liu , and C. Ring . 1993. “A Method of Constant Stimuli for Examining Heartbeat Detection: Comparison With the Brener‐Kluvitse and Whitehead Methods.” Psychophysiology 30, no. 6: 657–665. 10.1111/j.1469-8986.1993.tb02091.x. 8248457

[psyp70284-bib-0005] Brener, J. , and C. Ring . 2016. “Towards a Psychophysics of Interoceptive Processes: The Measurement of Heartbeat Detection.” Philosophical Transactions of the Royal Society, B: Biological Sciences 371, no. 1708: 20160015. 10.1098/rstb.2016.0015. PMC5062103 28080972

[psyp70284-bib-0006] Chari, P. , K. Kabra , D. Karinca , et al. 2020. “Diverse R‐PPG: Camera‐Based Heart Rate Estimation for Diverse Subject Skin‐Tones and Scenes.” *arXiv*. arXiv:2010.12769. 10.48550/arXiv.2010.12769.

[psyp70284-bib-0007] Cohen, J. 1988. “Set Correlation and Contingency Tables.” Applied Psychological Measurement 12, no. 4: 425–434. 10.1177/014662168801200410.

[psyp70284-bib-0008] Desmedt, O. , O. Luminet , and O. Corneille . 2018. “The Heartbeat Counting Task Largely Involves Non‐Interoceptive Processes: Evidence From Both the Original and an Adapted Counting Task.” Biological Psychology 138: 185–188. 10.1016/j.biopsycho.2018.09.004. 30218689

[psyp70284-bib-0009] Desmedt, O. , O. Luminet , P. Maurage , and O. Corneille . 2023. “Discrepancies in the Definition and Measurement of Human Interoception: A Comprehensive Discussion and Suggested Ways Forward.” Perspectives on Psychological Science 20: 17456916231191537. 10.1177/17456916231191537. 37642084

[psyp70284-bib-0010] Desmedt, O. , O. Luminet , M. Walentynowicz , and O. Corneille . 2023. “The New Measures of Interoceptive Accuracy: A Systematic Review and Assessment.” Neuroscience and Biobehavioral Reviews 153: 105388. 10.1016/j.neubiorev.2023.105388. 37708919

[psyp70284-bib-0011] Desmedt, O. , and O. Van den Bergh . 2024. “Beyond Interoceptive Accuracy: New Directions in Interoception Research.” Biological Psychology 189: 108800. 10.1016/j.biopsycho.2024.108800. 38631551

[psyp70284-bib-0012] Di Lernia, D. , G. Finotti , M. Tsakiris , G. Riva , and M. Naber . 2024. “Remote Photoplethysmography (rPPG) in the Wild: Remote Heart Rate Imaging via Online Webcams.” Behavior Research and Methods 56: 6904–6914. 10.3758/s13428-024-02398-0. PMC11362249 38632165

[psyp70284-bib-0013] Dotan, D. , F. Meyniel , and S. Dehaene . 2018. “On‐Line Confidence Monitoring During Decision Making.” Cognition 171: 112–121. 10.1016/j.cognition.2017.11.001. 29128659

[psyp70284-bib-0014] Faul, F. , E. Erdfelder , A.‐G. Lang , and A. Buchner . 2007. “G*Power 3: A Flexible Statistical Power Analysis Program for the Social, Behavioral, and Biomedical Sciences.” Behavior Research Methods 39, no. 2: 175–191. 10.3758/BF03193146. 17695343

[psyp70284-bib-0015] Folz, J. , M. Nikolić , and M. E. Kret . 2024. “Individual Differences in Interoception and Autistic Traits Share Altered Facial Emotion Perception, but Not Recognition Per Se.” Scientific Reports 14, no. 1: 19455. 10.1038/s41598-024-70299-5. 39169205 PMC11339312

[psyp70284-bib-0016] Füstös, J. , K. Gramann , B. M. Herbert , and O. Pollatos . 2013. “On the Embodiment of Emotion Regulation: Interoceptive Awareness Facilitates Reappraisal.” Social Cognitive and Affective Neuroscience 8, no. 8: 911–917. 10.1093/scan/nss089. 22933520 PMC3831556

[psyp70284-bib-0017] Gabriele, E. , R. Spooner , R. Brewer , and J. Murphy . 2022. “Dissociations Between Self‐Reported Interoceptive Accuracy and Attention: Evidence From the Interoceptive Attention Scale.” Biological Psychology 168: 108243. 10.1016/j.biopsycho.2021.108243. 34929353

[psyp70284-bib-0018] Garfinkel, S. N. , A. K. Seth , A. B. Barrett , K. Suzuki , and H. D. Critchley . 2015. “Knowing Your Own Heart: Distinguishing Interoceptive Accuracy From Interoceptive Awareness.” Biological Psychology 104: 65–74. 10.1016/j.biopsycho.2014.11.004. 25451381

[psyp70284-bib-0019] Gescheider, G. A. 2013. Psychophysics: The Fundamentals. Psychology Press.

[psyp70284-bib-0020] Herman, A. M. , C. Palmer , R. T. Azevedo , and M. Tsakiris . 2021. “Neural Divergence and Convergence for Attention to and Detection of Interoceptive and Somatosensory Stimuli.” Cortex 135: 186–206. 10.1016/j.cortex.2020.11.019. 33385747

[psyp70284-bib-0021] Jeganathan, J. , M. E. J. Campbell , N. Legrand , M. Allen , and M. Breakspear . 2025. “Aberrant Cardiac Interoception in Psychosis.” Schizophrenia Bulletin 51, no. 1: 208–216. 10.1093/schbul/sbae078. PMC11661957 38788050

[psyp70284-bib-0022] Katkin, E. S. , J. Blascovich , and S. Goldband . 1981. “Empirical Assessment of Visceral Self‐Perception: Individual and Sex Differences in the Acquisition of Heartbeat Discrimination.” Journal of Personality and Social Psychology 40, no. 6: 1095–1101. 10.1037/0022-3514.40.6.1095. 7264877

[psyp70284-bib-0023] Khalsa, S. S. , R. Adolphs , O. G. Cameron , et al. 2018. “Interoception and Mental Health: A Roadmap.” Biological Psychiatry: Cognitive Neuroscience and Neuroimaging, Interoception and Mental Health 3, no. 6: 501–513. 10.1016/j.bpsc.2017.12.004. PMC6054486 29884281

[psyp70284-bib-0024] Kleiner, M. , D. Brainard , and D. Pelli . 2007. What's New in Psychtoolbox‐3? Vol. 89. Pion Ltd.

[psyp70284-bib-0025] Knapp, K. , C. Ring , and J. Brener . 1997. “Sensitivity to Mechanical Stimuli and the Role of General Sensory and Perceptual Processes in Heartbeat Detection.” Psychophysiology 34, no. 4: 467–473. 10.1111/j.1469-8986.1997.tb02391.x. 9260500

[psyp70284-bib-0026] Koo, T. K. , and M. Y. Li . 2016. “A Guideline of Selecting and Reporting Intraclass Correlation Coefficients for Reliability Research.” Journal of Chiropractic Medicine 15, no. 2: 155–163. 10.1016/j.jcm.2016.02.012. 27330520 PMC4913118

[psyp70284-bib-0027] Körmendi, J. , E. Ferentzi , T. Petzke , V. Gál , and F. Köteles . 2023. “Do We Need to Accurately Perceive Our Heartbeats? Cardioceptive Accuracy and Sensibility Are Independent From Indicators of Negative Affectivity, Body Awareness, Body Image Dissatisfaction, and Alexithymia.” PLoS One 18, no. 7: e0287898. 10.1371/journal.pone.0287898. 37406011 PMC10321613

[psyp70284-bib-0028] Larsson, D. E. O. , G. Esposito , H. D. Critchley , Z. Dienes , and S. N. Garfinkel . 2021. “Sensitivity to Changes in Rate of Heartbeats as a Measure of Interoceptive Ability.” Journal of Neurophysiology 126, no. 5: 1799–1813. 10.1152/jn.00059.2021. 34669507

[psyp70284-bib-0029] Legrand, N. , N. Nikolova , C. Correa , et al. 2022. “The Heart Rate Discrimination Task: A Psychophysical Method to Estimate the Accuracy and Precision of Interoceptive Beliefs.” Biological Psychology 168: 108239. 10.1016/j.biopsycho.2021.108239. 34902450

[psyp70284-bib-0030] Lernia, D. D. , G. Finotti , M. Tsakiris , G. Riva , and M. Naber . 2022. “Remote Photoplethysmography (rPPG) in the Wild: Remote Heart Rate Imaging via Online Webcams.” *PsyArXiv*. 10.31234/osf.io/v89zn. PMC11362249 38632165

[psyp70284-bib-0031] Leys, C. , C. Ley , O. Klein , P. Bernard , and L. Licata . 2013. “Detecting Outliers: Do Not Use Standard Deviation Around the Mean, Use Absolute Deviation Around the Median.” Journal of Experimental Social Psychology 49, no. 4: 764–766. 10.1016/j.jesp.2013.03.013.

[psyp70284-bib-0032] Li, P. , Q. Lu , Q. Wu , X. Liu , and Y. Wu . 2021. “What Makes an Elite Shooter and Archer? The Critical Role of Interoceptive Attention.” Frontiers in Psychology 12: 666568. 10.3389/fpsyg.2021.666568. 34177723 PMC8219872

[psyp70284-bib-0033] Liu, X. , G. Narayanswamy , A. Paruchuri , et al. 2023. “rPPG‐Toolbox: Deep Remote PPG Toolbox.” Advances in Neural Information Processing Systems 36: 68485–68510.

[psyp70284-bib-0034] Maister, L. , T. Tang , and M. Tsakiris . 2017. “Neurobehavioral Evidence of Interoceptive Sensitivity in Early Infancy.” eLife 6: e25318. 10.7554/eLife.25318. 28784203 PMC5548485

[psyp70284-bib-0035] Mason, J. W. , D. J. Ramseth , D. O. Chanter , T. E. Moon , D. B. Goodman , and B. Mendzelevski . 2007. “Electrocardiographic Reference Ranges Derived From 79,743 Ambulatory Subjects.” Journal of Electrocardiology 40, no. 3: 228–234.e8. 10.1016/j.jelectrocard.2006.09.003. 17276451

[psyp70284-bib-0036] Matthews, G. , J. Szalma , A. R. Panganiban , C. Neubauer , and J. S. Warm . 2013. “Profiling Task Stress With the Dundee Stress State Questionnaire.” In Psychology of Stress: New Research, 49–91. Nova Science Publishers, Incorporated.

[psyp70284-bib-0037] Mehling, W. E. , M. Acree , A. Stewart , J. Silas , and A. Jones . 2018. “The Multidimensional Assessment of Interoceptive Awareness, Version 2 (MAIA‐2).” PLoS One 13, no. 12: e0208034. 10.1371/journal.pone.0208034. 30513087 PMC6279042

[psyp70284-bib-0038] Mehling, W. E. , C. Price , J. J. Daubenmier , M. Acree , E. Bartmess , and A. Stewart . 2012. “The Multidimensional Assessment of Interoceptive Awareness (MAIA).” PLoS One 7, no. 11: e48230. 10.1371/journal.pone.0048230. 23133619 PMC3486814

[psyp70284-bib-0039] Moffatt, J. , G. Finotti , and M. Tsakiris . 2024. “With Hand on Heart: A Cardiac Rubber Hand Illusion.” Biological Psychology 186: 108756. 10.1016/j.biopsycho.2024.108756. 38280444

[psyp70284-bib-0040] Murphy, J. 2024. “Interoception: Where Do We Go From Here?” Quarterly Journal of Experimental Psychology 77, no. 2: 223–229. 10.1177/17470218231172725. PMC10798007 37082986

[psyp70284-bib-0041] Murphy, J. , R. Brewer , D. Plans , S. S. Khalsa , C. Catmur , and G. Bird . 2020. “Testing the Independence of Self‐Reported Interoceptive Accuracy and Attention.” Quarterly Journal of Experimental Psychology 73, no. 1: 115–133. 10.1177/1747021819879826. 31519137

[psyp70284-bib-0042] Nowara, E. M. , D. McDuff , and A. Veeraraghavan . 2020. “A Meta‐Analysis of the Impact of Skin Tone and Gender on Non‐Contact Photoplethysmography Measurements.” In Proceedings of the IEEE/CVF Conference on Computer Vision and Pattern Recognition Workships, 284–285. IEEE. https://openaccess.thecvf.com/content_CVPRW_2020/html/w19/Nowara_A_Meta‐Analysis_of_the_Impact_of_Skin_Tone_and_Gender_CVPRW_2020_paper.html.

[psyp70284-bib-0043] Palmer, C. , V. Ainley , and M. Tsakiris . 2019. “Fine Tuning Your Heart: A Novel Method for Measuring Interoceptive Accuracy.” *PsyArXiv*. 10.31234/osf.io/qz7r9.

[psyp70284-bib-0044] Pirzada, P. , A. Wilde , G. H. Doherty , and D. Harris‐Birtill . 2023. “Remote Photoplethysmography (rPPG): A State‐of‐the‐Art Review.” *medRxiv*. 2023.10.12.23296882. 10.1101/2023.10.12.23296882.

[psyp70284-bib-0045] Plans, D. , S. Ponzo , D. Morelli , et al. 2021. “Measuring Interoception: The Phase Adjustment Task.” Biological Psychology 165: 108171. 10.1016/j.biopsycho.2021.108171. 34411620

[psyp70284-bib-0046] Ponzo, S. , D. Morelli , C. Suksasilp , M. Cairo , and D. Plans . 2021. “Measuring Interoception: The CARdiac Elevation Detection Task.” Frontiers in Psychology 12: 712896. 10.3389/fpsyg.2021.712896. 34489814 PMC8416769

[psyp70284-bib-0047] Ree, M. J. , D. French , C. MacLeod , and V. Locke . 2008. “Distinguishing Cognitive and Somatic Dimensions of State and Trait Anxiety: Development and Validation of the State‐Trait Inventory for Cognitive and Somatic Anxiety (STICSA).” Behavioural and Cognitive Psychotherapy 36, no. 3: 313–332. 10.1017/S1352465808004232.

[psyp70284-bib-0048] Ring, C. , and J. Brener . 2018. “Heartbeat Counting Is Unrelated to Heartbeat Detection: A Comparison of Methods to Quantify Interoception.” Psychophysiology 55, no. 9: e13084. 10.1111/psyp.13084. 29633292

[psyp70284-bib-0049] Savage, H. , and S. Garfinkel . 2025. Making Sense of Sensation: A Model of Interoceptive Attribution and Appraisal With Clinical Applications. OSF. 10.31234/osf.io/bsma9_v1.

[psyp70284-bib-0050] Schandry, R. 1981. “Heart Beat Perception and Emotional Experience.” Psychophysiology 18, no. 4: 483–488. 10.1111/j.1469-8986.1981.tb02486.x. 7267933

[psyp70284-bib-0051] Sierra, M. , and G. E. Berrios . 2000. “The Cambridge Depersonalisation Scale: A New Instrument for the Measurement of Depersonalisation.” Psychiatry Research 93, no. 2: 153–164. 10.1016/S0165-1781(00)00100-1. 10725532

[psyp70284-bib-0052] Smith, R. , J. S. Feinstein , R. Kuplicki , et al. 2021. “Perceptual Insensitivity to the Modulation of Interoceptive Signals in Depression, Anxiety, and Substance Use Disorders.” Scientific Reports 11, no. 1: 1. 10.1038/s41598-021-81307-3. 33483527 PMC7822872

[psyp70284-bib-0059] Spooner, R. , J. M. Bird , R. Clemente , et al. 2024. “No Differences Between Remote and Laboratory‐Based Testing of Cardiac Interoceptive Accuracy Using the Phase Adjustment Task.” Scientific Reports 14, no. 1: 28524. 10.1038/s41598-024-79125-4. 39557961 PMC11574115

[psyp70284-bib-0053] Strauss, M. E. , and G. T. Smith . 2009. “Construct Validity: Advances in Theory and Methodology.” Annual Review of Clinical Psychology 5, no. 1: 1–25. 10.1146/annurev.clinpsy.032408.153639. PMC2739261 19086835

[psyp70284-bib-0054] Suksasilp, C. , and S. N. Garfinkel . 2022. “Towards a Comprehensive Assessment of Interoception in a Multi‐Dimensional Framework.” Biological Psychology 168: 108262. 10.1016/j.biopsycho.2022.108262. 35026353

[psyp70284-bib-0055] van der Kooij, K. M. , and M. Naber . 2019. “An Open‐Source Remote Heart Rate Imaging Method With Practical Apparatus and Algorithms.” Behavior Research Methods 51, no. 5: 2106–2119. 10.3758/s13428-019-01256-8. 31152386 PMC6797647

[psyp70284-bib-0056] Whitehead, W. E. , V. M. Drescher , P. Heiman , and B. Blackwell . 1977. “Relation of Heart Rate Control to Heartbeat Perception.” Biofeedback and Self‐Regulation 2, no. 4: 371–392. 10.1007/BF00998623. 612350

[psyp70284-bib-0057] Whitely, S. E. 1983. “Construct Validity: Construct Representation Versus Nomothetic Span.” Psychological Bulletin 93, no. 1: 179–197. 10.1037/0033-2909.93.1.179.

[psyp70284-bib-0058] Wittkamp, M. F. , K. Bertsch , C. Vögele , and A. Schulz . 2018. “A Latent State‐Trait Analysis of Interoceptive Accuracy.” Psychophysiology 55, no. 6: e13055. 10.1111/psyp.13055. 29337347

