# Supplementary material for: Validity and Reliability of the Heart Rate Matching Task: A Novel Measure of Heart Rate Estimation
Source: Psychophysiology. 2026 Apr 2;63(4):e70284. doi: 10.1111/psyp.70284 (PMC13047253; doi:10.1111/psyp.70284)
Supplement: Supplementary file 1 — Appendix S1: psyp70284‐sup‐0001‐supinfo.docx. [file PSYP-63-e70284-s001.docx]

Supplementary Materials

Validity and reliability of the Heart Rate Matching task, a novel measure of heart rate estimation

Jamie A. Moffatt^1,2^, Markus R. Tünte^1,^ ^3^, Mariana Von Mohr^1^, & Manos Tsakiris^1^

^1^Lab of Action & Body, Department of Psychology, Royal Holloway, University of London

^2^Department of Psychology, Durham University

^3^Department of Developmental and Educational Psychology, University of Vienna

1. **Experiment 1, heart rate estimated with rPPG**

Heart Rate Matching (HRM) metrics in Experiment 1 were calculated by estimating heart rate using Electrocardiography (ECG), the gold standard for measuring heart rate. During this experiment, heart rate was also estimated from videos of the participant’s face using remote photoplethysmography (rPPG) methods. The following analysis presents data on HRM performance and associations between HRM and Interoceptive Accuracy if the HRM metrics were calculated with the rPPG methodology.

- 1. **HRM Performance.**

Averages for the HRM are reported in Table S1. Bias scores (reported heart rate minus actual heart rate) were under-estimated by 12.25 beats-per-minute (BPM) when calculated with heart rate estimated from rPPG.

Table S1. Heart Rate Matching, Experiment 1

|  | N | Reported BPM | Target BPM | Bias (BPM) | Abs Bias (BPM) | Variance | Confidence |
| --- | --- | --- | --- | --- | --- | --- | --- |
| HRM | 41 | 68.6 | 80.81 | -12.25 | 16.34 | 8.92 | 49.8 |
| Note. Target BPM represents heart rate calculated with remote photplethysmography (rPPG). Bias, Absolute Bias and Variance are all calculated with rPPG estimated heart rate. HRM = Heart Rate Matching, N = sample size, BPM = beats-per-minute. | | | | | | | |

- 1. **Associations between HRM and Interoceptive Accuracy**

Figure S1 presents correlations between variables on the HRM, and with IAcc derived from the Heartbeat Counting and Heartbeat Discrimination tasks, if the metrics are calculated with rPPG methods. The only difference in terms of significant effects between Figure S1 and the matching figure in the main manuscript (Figure 2) is the correlation between Bias and Target BPM, which is no longer significant if calculated with rPPG methods. The correlation between Bias and IAcc derived from Heartbeat Counting is slightly weaker with Bias derived from rPPG (r = 0.15) than with ECG (r = 0.21), and the correlation between Bias and IAcc derived from Heartbeat Discrimination is slighty weaker if Bias is derived from rPPG (r = -0.04) than with ECG (r = -0.05).


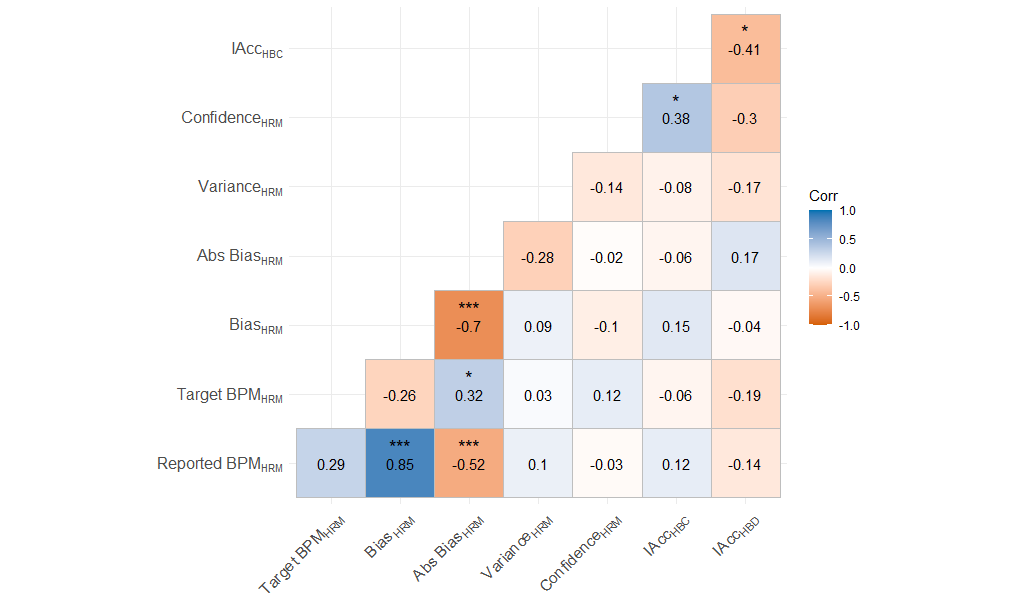


Figure S1. Associations between Matching, Counting and Discrimination tasks when Matching variables are calculated with remote photoplethysmography (rPPG). BPM = beats-per-minute, HRM = Heart Rate Matching, HBD = Heartbeat Discrimination, HBC = Heartbeat Counting, IAcc = Interoceptive Accuracy.

1. **Experiment 2, Correlations with Questionnaires**

Here we report all correlations between outcome variables of the Heart Rate Matching, the Audio Rate Matching task and the Heartbeat Counting tasks with the questionnaires recorded in Experiment 2: the Multi-dimensional Assessment of Interoceptive Awareness, Version 2 (MAIA-2), the Interoceptive Accuracy Scale (IAS) and Interoceptive Attention Scale (IATS), the Cambridge Depersonalisation Scale (CDS), the Toronto Alexithymia Scale (TAS) and the State-Trait Inventory for Cognitive and Somatic Anxiety (STICSA).

- 1. **Correlations between HRM and Interoceptive Awareness**


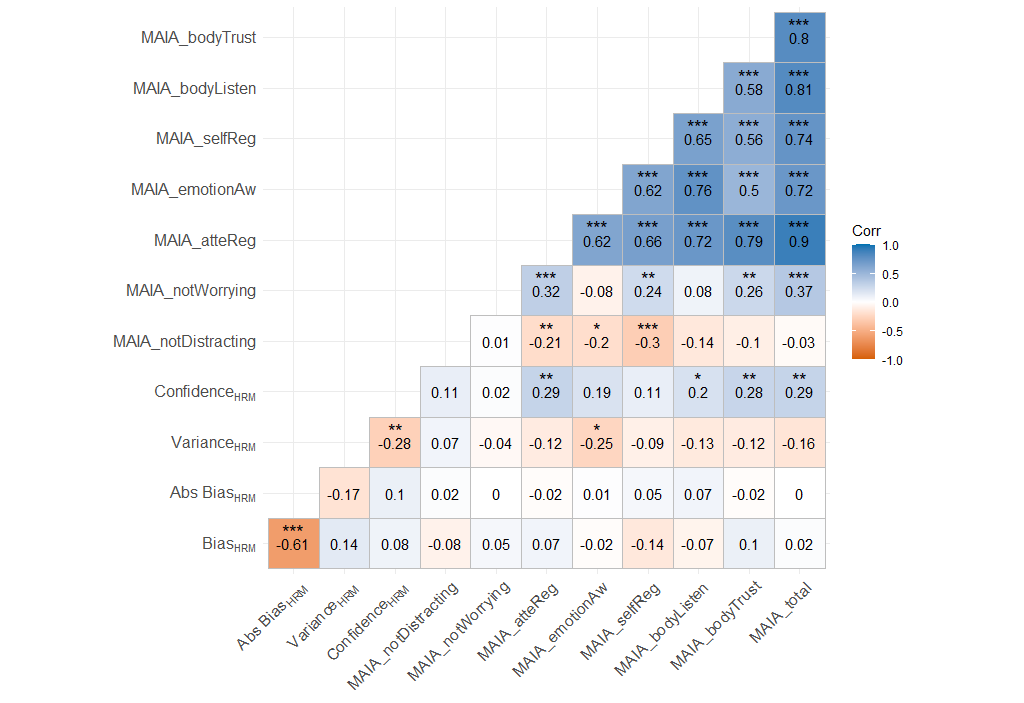


Figure S2. Heart Rate Matching & MAIA subscale associations. Correlations are Spearman correlations. HRM = Heart Rate Matching, notDistracting = Not Distracting subscale, notWorrying = Not Worrying subscale, atteReg = Attention Regulation subscale, emotionAw = Emotion Awareness subscale, selfReg = Self Regulation subscale. bodyListen = Body Listening subscale, bodyTrust = Body Trust subscale, total = Total score. *p<0.05, **p<0.01, ***p<0.001.

**2.2. Correlations between ARM and Interoceptive Awareness**


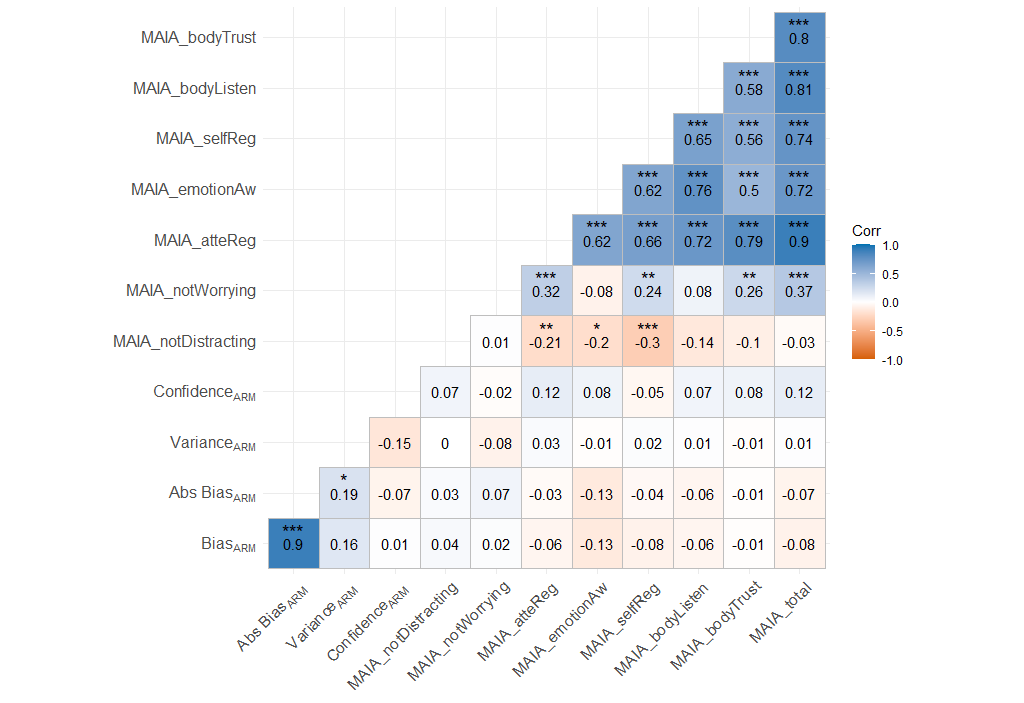


Figure S3. Audio Rate Matching & MAIA subscale associations. Correlations are Spearman correlations. ARM = Audio Rate Matching, notDistracting = Not Distracting subscale, notWorrying = Not Worrying subscale, atteReg = Attention Regulation subscale, emotionAw = Emotion Awareness subscale, selfReg = Self Regulation subscale. bodyListen = Body Listening subscale, bodyTrust = Body Trust subscale, total = Total score. *p<0.05, **p<0.01, ***p<0.001.

- 1. **Correlations with Interoceptive Accuracy and Interoceptive Attention**


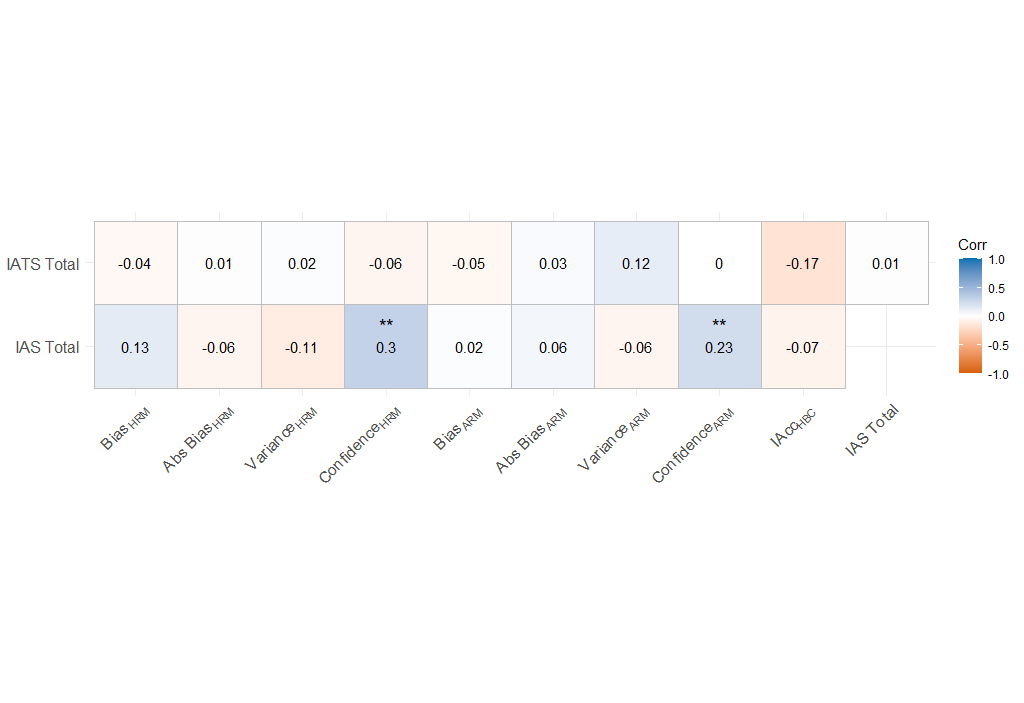


Figure S4. Association with Interoceptive Accuracy Scale & Interoception Attention Scale. Correlations are Spearman correlations. HRM = Heart Rate Matching, ARM = Audio Rate Matching, IAcc = Interoceptive Accuracy, HBC = Heartbeat Counting, IAS = Interoceptive Accuracy Scale total score, IATS = Interoceptive Attention Scale total score. *p<0.05, **p<0.01, ***p<0.001.

- 1. **Correlations with Dissociation**


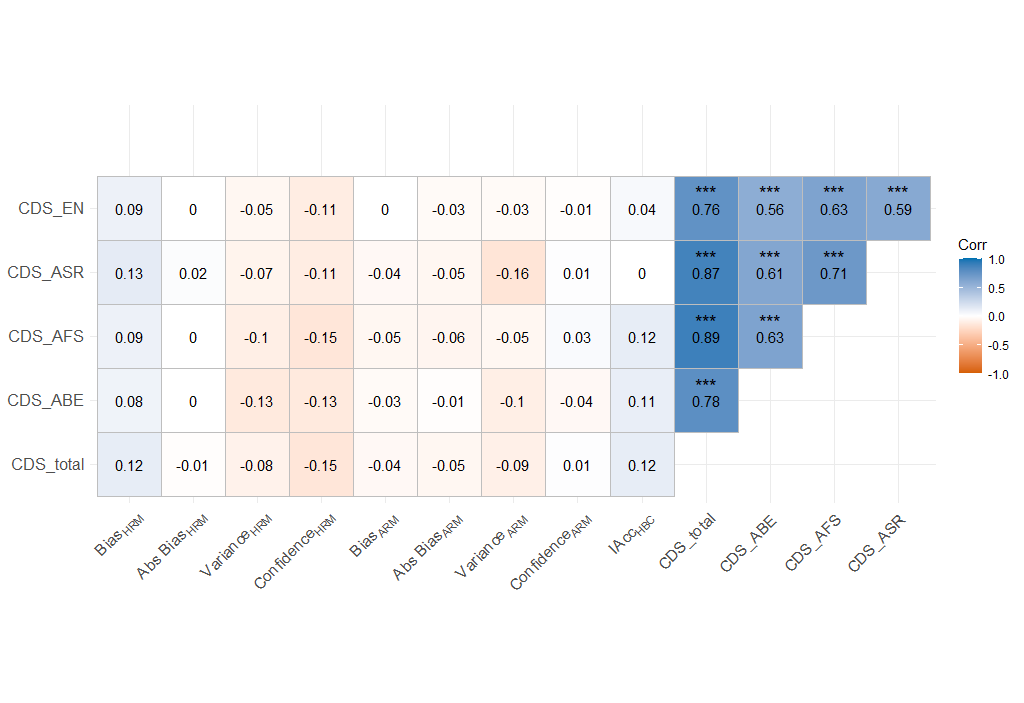


Figure S5. Correlations between CDS, HRM, ARM and HBC variables. Correlations are Spearman correlations. HRM = Heart Rate Matching, ARM = Audio Rate Matching, IAcc = Interoceptive Accuracy, HBC = Heartbeat Counting, CDS = Cambridge Depersonalisation Scale, CDS_ABE = Anomalous Body Experiences subscale, CDS_AFS = Alienation From Surroundings subscale, CDS_EN = Emotional Numbing subscale, CDS_ASR = Anomalous Subjective Recall subscale. *p<0.05, **p<0.01, ***p<0.001.

- 1. **Correlations with Alexithymia**


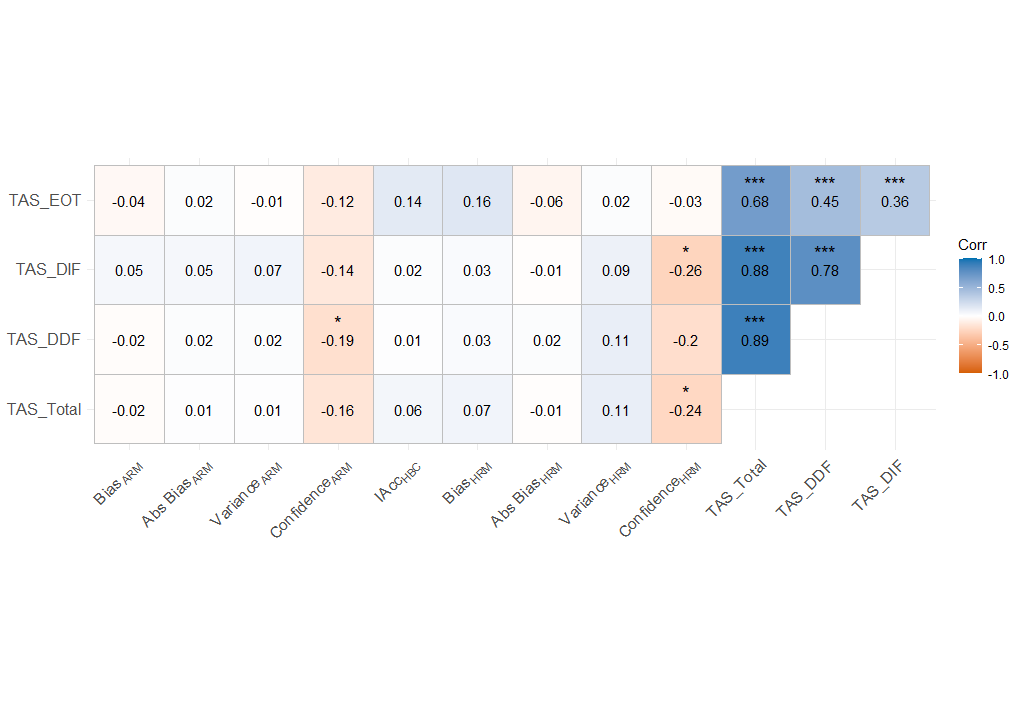


Figure S6. Correlations with Toronto Alexithymia Scale (TAS). Correlations are Spearman correlations. HRM = Heart Rate Matching, ARM = Audio Rate Matching, IAcc = Interoceptive Accuracy, HBC = Heartbeat Counting, TAS_DDF = Difficulty Describing Feelings subscale. TAS_DIF = Difficulty Identifying Feelings subscale. TAS_EOT = Externally-Oriented Thinking subscale. *p<0.05, **p<0.01, ***p<0.001.

- 1. **Correlations with Anxiety**


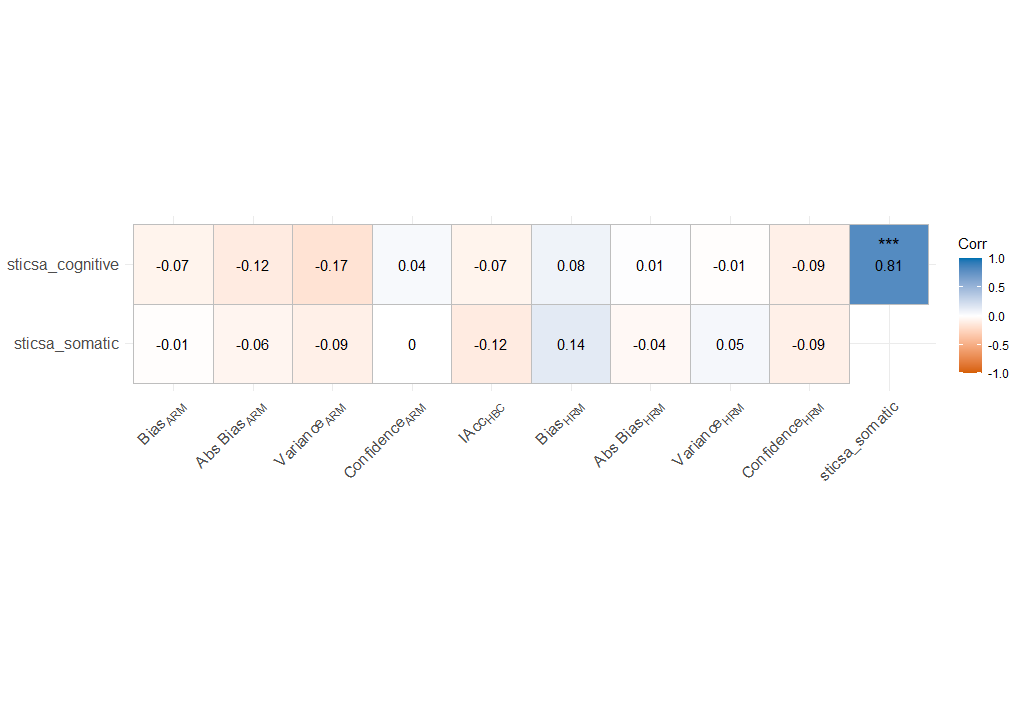


Figure S7. Correlations between STICSA, HRM, ARM and HBC variables. Correlations are Spearman correlations. HRM = Heart Rate Matching, ARM = Audio Rate Matching, IAcc = Interoceptive Accuracy, HBC = Heartbeat Counting. *p<0.05, **p<0.01, ***p<0.001.

**3. Comparison across Experiments**

Across three experiments we have investigated validity of the HRM. In particular, we focused on whether Bias was related to interoceptive ability across a range or measures. We found some evidence for a positive relationship between bias and heartbeat counting scores in Experiment 2 but failed to find a significant correlation with other measures. However, a non-significant result does not indicate that there is no relationship between the measures used (Harms & Lakens, 2018). Instead, the sample size might have not been sufficient to detect smaller effects. Given that the relationship between measures of interoceptive accuracy is oftentimes small, here we want to provide additional information for the interpretation of the relationship between Bias and interoceptive accuracy.

We conducted a number of equivalence tests for the correlation between bias and measures of interoceptive accuracy using the r-package TOSTER (Caldwell, 2022). In brief, equivalence tests help at contrasting whether a result, given an equivalence bound, can be used to interpret evidence as for an effect (the 95% CIs only include the equivalence bound, but not 0), against an effect (the 95% CIs only include 0, but not the equivalence bound), or being undecided (the 95% CIs include both 0 and the equivalence bound). An equivalence bound is an effect size termed meaningful in the context of the measures. Here, we used the correlation between Bias and the heartbeat counting score found in Experiment 2 (*r* = .26) as equivalence bound, as we consider it the strongest evidence collected across our studies due to the larger sample size. Then, we tested whether the correlation found between measures of interoceptive accuracy and bias in the other experiments can discriminate between a null effect and an effect of at least *r* = .26. We find that across all measures (Figure 11) we cannot rule out that the relationship between measures of interoceptive ability and bias is at least *r* = .26 (*p_hct_exp1_* = .787, *p_hdt_exp1_* = .107, *p_hrd_exp3.1_* = .711, *p_hrd_exp3.2_* = .671).


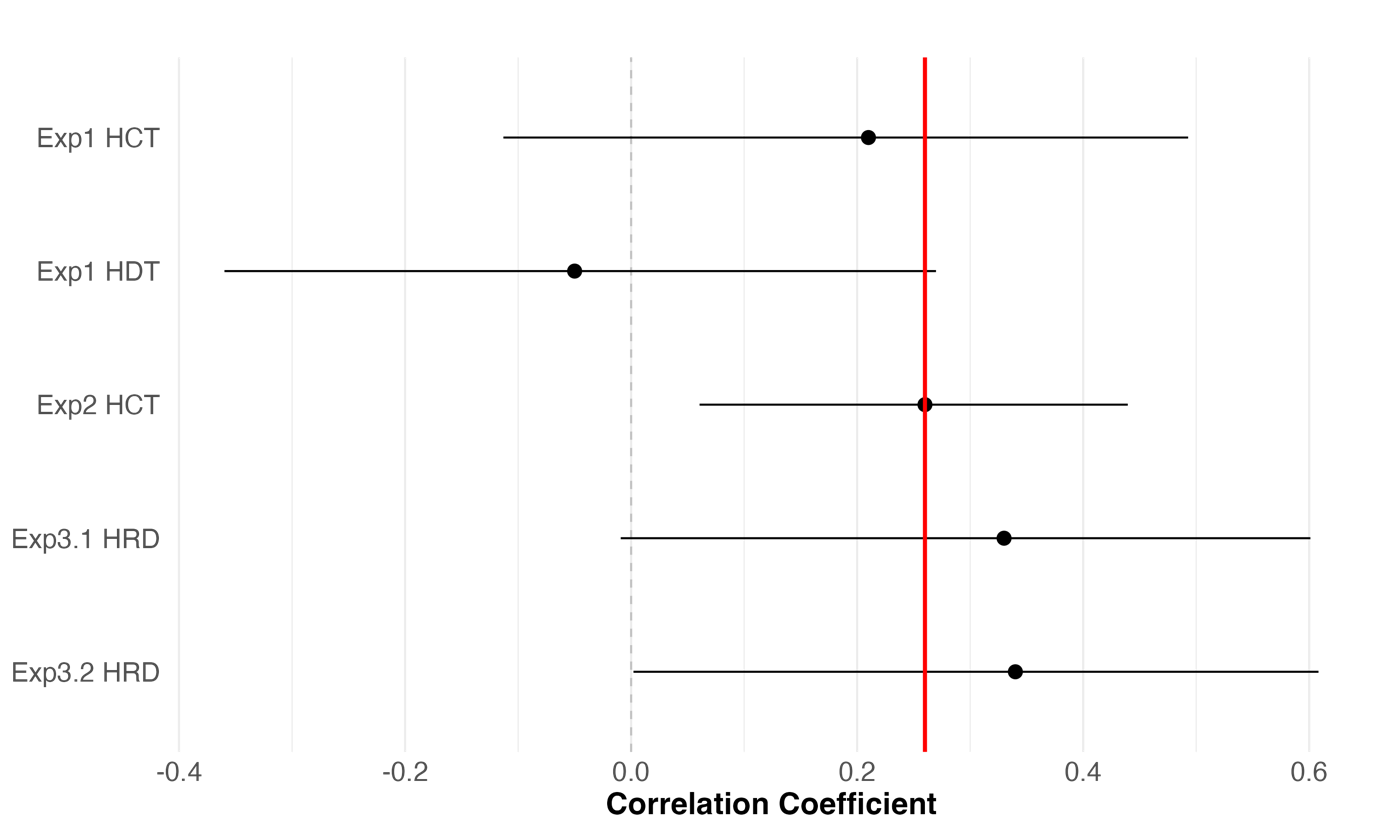


*Figure S8. Forest plot showing correlation coefficient and 95% confidence intervals between Bias and measures of interoceptive ability across experiments. The red line indicates the equivalence bound (r = .26). HCT = Heartbeat Counting task, HDT = Heartbeat Discrimination task, HRD = Heart Rate discrimination task, Exp = Experiment.*

In sum, we find that for the nonsignificant correlations reported above we cannot rule out that the relationship between Bias and measures of interoceptive ability is at least *r* = .26. This is potentially due to the smaller sample size in those samples. In the largest sample (Experiment 2) we find a significant result. Given the pattern of results and point estimates found it is possible that Bias is weakly correlated with other measures of interoceptive ability, which is in line with correlations commonly found between measures of interoceptive accuracy (Hickman et al., 2020; Legrand et al., 2022).
